# Large-scale annotation of biochemically relevant pockets and tunnels in cognate enzyme–ligand complexes

**DOI:** 10.1186/s13321-024-00907-z

**Published:** 2024-10-15

**Authors:** O. Vavra, J. Tyzack, F. Haddadi, J. Stourac, J. Damborsky, S. Mazurenko, J. M. Thornton, D. Bednar

**Affiliations:** 1 Loschmidt Laboratories, Department of Experimental Biology and RECETOX, Faculty of Science, Masaryk University, Kamenice 5/A13, 625 00 Brno, Czech Republic; 2 International Clinical Research Center, St. Anne’s University Hospital Brno, Pekařská 53, 656 91 Brno, Czech Republic; 3 European Molecular Biology Laboratory, European Bioinformatics Institute (EMBL-EBI), Wellcome Trust GenomeCampus, Cambridge, CB10 1SD UK

**Keywords:** Bottleneck, Cognate ligand, Cavity, Enzyme, Tunnel, Machine learning, Pocket, Transport

## Abstract

Tunnels in enzymes with buried active sites are key structural features allowing the entry of substrates and the release of products, thus contributing to the catalytic efficiency. Targeting the bottlenecks of protein tunnels is also a powerful protein engineering strategy. However, the identification of functional tunnels in multiple protein structures is a non-trivial task that can only be addressed computationally. We present a pipeline integrating automated structural analysis with an *in-house* machine-learning predictor for the annotation of protein pockets, followed by the calculation of the energetics of ligand transport via biochemically relevant tunnels. A thorough validation using eight distinct molecular systems revealed that CaverDock analysis of ligand un/binding is on par with time-consuming molecular dynamics simulations, but much faster. The optimized and validated pipeline was applied to annotate more than 17,000 cognate enzyme–ligand complexes. Analysis of ligand un/binding energetics indicates that the top priority tunnel has the most favourable energies in 75% of cases. Moreover, energy profiles of cognate ligands revealed that a simple geometry analysis can correctly identify tunnel bottlenecks only in 50% of cases. Our study provides essential information for the interpretation of results from tunnel calculation and energy profiling in mechanistic enzymology and protein engineering. We formulated several simple rules allowing identification of biochemically relevant tunnels based on the binding pockets, tunnel geometry, and ligand transport energy profiles.

**Scientific contributions**

The pipeline introduced in this work allows for the detailed analysis of a large set of protein–ligand complexes, focusing on transport pathways. We are introducing a novel predictor for determining the relevance of binding pockets for tunnel calculation. For the first time in the field, we present a high-throughput energetic analysis of ligand binding and unbinding, showing that approximate methods for these simulations can identify additional mutagenesis hotspots in enzymes compared to purely geometrical methods. The predictor is included in the supplementary material and can also be accessed at https://github.com/Faranehhad/Large-Scale-Pocket-Tunnel-Annotation.git. The tunnel data calculated in this study has been made publicly available as part of the ChannelsDB 2.0 database, accessible at https://channelsdb2.biodata.ceitec.cz/.

**Supplementary Information:**

The online version contains supplementary material available at 10.1186/s13321-024-00907-z.

## Introduction

Enzymes are biological catalysts that can accelerate chemical reactions, which makes them essential for every living cell. These chemical reactions occur in the active site, which consists of residues with specific physicochemical properties. Active sites can be found either in clefts on the surface of an enzyme or buried inside a cavity shielded from the outer environment. In the latter case, the active site cavity is connected with the surface by access tunnels to enable the passage of ligands, small molecules that interact with the enzyme [1]. This encompasses the exchange of reactant and product molecules or the binding of cofactors. The tunnels also impact the activity and specificity of the enzyme by restricting access to the active site for unfavourable molecules [2]. The introduction of mutations in protein tunnels and channels can affect activity, specificity, promiscuity, enantioselectivity, and stability [3, 4].

Several computational tools were developed for the detection of important cavities and pockets, e.g., Fpocket [5], CASTp [6], and P2Rank [7]. These tools rank all the pockets found in a protein structure by their scoring functions and select the best potential binding pocket for the user. To improve the reliability of the selection, one can use annotations found in structure databases [8, 9]. Unfortunately, these annotations are available only for a limited number of enzymes. The selection of the functionally relevant pocket is also crucial for the calculation of access tunnels. However, currently there is no tool available that would predict the suitability of a pocket for this purpose.

To identify tunnels in enzymes, one may use tools such as CAVER [10], MOLE [11] or MOLAXIS [12]. Similarly, with pocket calculation, these tools can detect multiple tunnels and also provide ways to rank them based on their geometrical properties. In many proteins with buried active sites, multiple tunnels can be identified, which makes it difficult to decide which tunnel is biochemically relevant. This crucial decision could be greatly supported by a large-scale analysis of protein structures. Previous efforts in this matter focused purely on finding tunnels in enzymes [13, 14]. While these studies proved that tunnels appear in all enzyme classes, they did not define how to recognise biochemically relevant tunnels.

The classical computational approach to studying the biological relevance of tunnels is to simulate the interactions between a protein and a ligand with methods based on molecular dynamics [15]. Unfortunately, this time-demanding type of simulation is not feasible for large datasets. More recent tools, such as CaverDock [16], GPathFinder [17], or ART-RRT [18], employ various approximations to simulate ligand transport in short computational times and provide valuable information about the energy profile of the process. These tools are gaining popularity [19] and have successfully been used for screening and identifying novel drugs [20, 21] and engineering proteins [22–26].

In this study, we present a novel strategy for annotating pocket relevance for tunnel calculation and assign biochemical relevance of tunnels based on ligand transport and binding energies. With the growing number of available protein structures [27] and models [28], automatic annotation of binding pockets and tunnels without the dependency on residue annotations would be of great use. Based on the premise that substrate and product molecules are present in relevant pockets in enzyme structures, we created a dataset independent of annotations. We selected experimentally derived enzyme structures with bound molecules that were similar to cognate ligands, i.e., ligands that potentially bind or react with a given enzyme. For this purpose, we used a previously published dataset of enzyme cognate ligand pairs [29–31], which we updated and utilized for structural analyses of pockets and tunnels in this study. We then developed a pipeline combining machine learning, the geometrical analysis of tunnels, and the energy profiling of transported ligands. The pipeline was then validated against molecular dynamics simulations and applied to the large-scale dataset with more than 17,000 protein structures.

## Methods

**Fig. 1:**
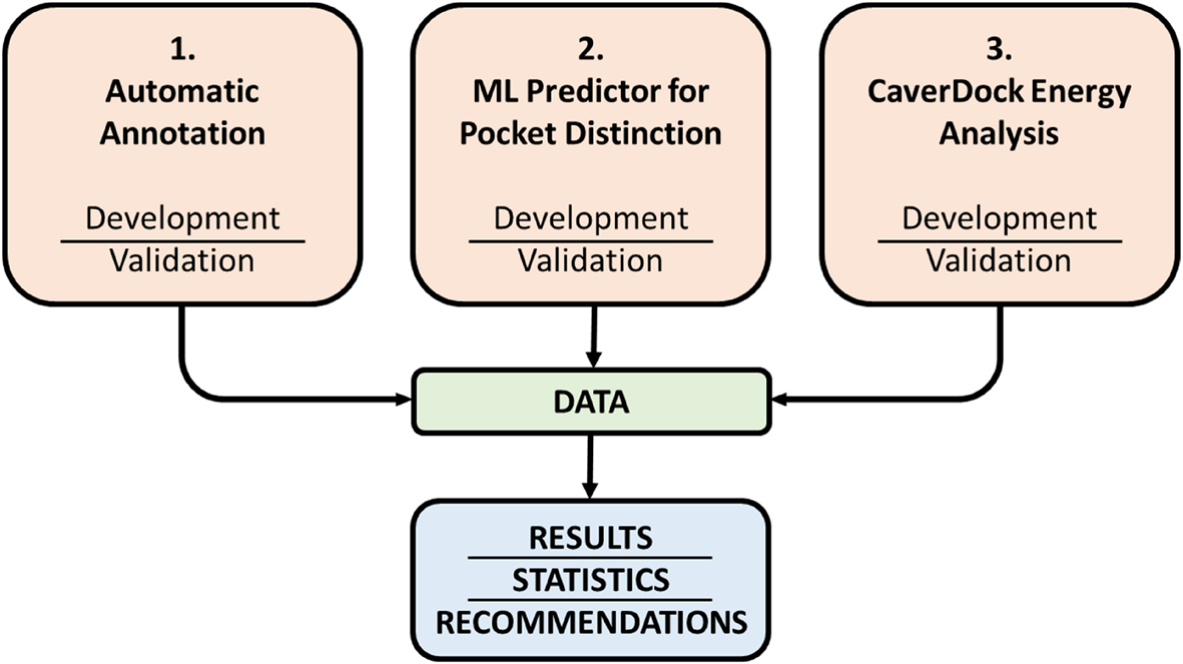
The overview of the pipeline developed in this study. The pipeline consists of three steps: (i) automatic annotation of enzyme–cognate ligand complexes with computational tools, (ii) classification of ligand binding pocket by machine learning (ML) predictor, and (iii) analysis of ligand transport through enzyme tunnels with CaverDock

The study used data collected from the publication by Tyzack et al*.* [31] and updated it for the purposes of our study. After filtering the original dataset, we analysed 17,092 unique protein–ligand pairs (Table 1) The data consists of enzyme–ligand complexes ranked by the similarity of the bound ligand with the cognate ligand from the KEGG [32] database calculated by the PARITY algorithm [31]. To process the data, we designed an automatic pipeline which consists of three parts (Fig. 1): (i) automatic annotation of enzyme–cognate ligand complexes by computational tools (ii) classification of the main binding pocket of the enzyme to buried or surface pocket by machine learning (ML) predictor, and (iii) the energetical analysis of ligand un/binding by CaverDock (Fig. 1). Each part of the pipeline was separately tested and validated. The data provided from all three parts were combined and analysed in the later part of the study. Here we provide a summary of the methodology behind the pipeline. The detailed description of each step with used parameters is part of the supplementary material.

**Table 1:** The summary of the pipeline proposed in this study and the dataset sizes at various stages of the pipeline execution

Pipeline	Items	Number of cases
1. Automatic annotation	Protein–ligand pairs	35,882
	Unique PDBs	17,092
	Ligand missing in the biological unit	193
	Ligand not present in PDB	133
	Ligand not present in any pocket	1058
	Pocket calculation errors	337
	Successfully calculated pockets	15,697
	Proteins with annotation in CSA and Uniprot	11,046
	Proteins without annotation	4651
	Selected pockets with matching annotated residues	8350
	No matching residues in the selected pocket	2696
	No tunnels found by Caver	526
	Tunnel calculation errors	739
	Successfully calculated tunnels	14,432
2. Machine Learning predictor for pocket annotation	Buried pockets without tunnels	508
	Borderline pockets without tunnels	160
	Surface pockets without tunnels	597
	Buried pockets with calculated tunnels	3552
	Borderline pockets with calculated tunnels	3178
	Surface pockets with calculated tunnels	7702
3. Energy profiles	Protein–ligand pairs for CaverDock calculations	14,432
	Unfinished CaverDock calculations	1274
	Successfully completed CaverDock jobs	13,158
	Successfully calculated energy profiles	29,693

### Automatic annotation

At the beginning of the pipeline, the biological unit is collected for each enzyme in the dataset [27]. The structures were processed to remove all ligand molecules, while known cofactors [5, 33] were kept in the structure. Next, we calculated pockets in the structure with Fpocket 2 [5] and selected the main pocket based on the location of the bound ligand structurally related to the cognate ligand of the enzyme. The selected pocket was used to define the starting point for tunnel detection using CAVER 3.02 [10]. The automatic annotation part of the pipeline was validated in two ways. First, we collected annotations from Swiss-Prot, UniProtKB [8], and CSA [9], together with Fpocket and druggability scores calculated for all pockets by Fpocket 2. This data was used to observe whether the selected main pocket contained annotated residues important for the function of the enzyme and to analyse if the main pocket had the best-predicted scores. Second, we studied the impact of the ligand presence in protein structures to determine the changes in tunnel properties. By using the REST API in PDBe [27] and RCSB [34], we collected 2904 pairs of protein–ligand complexes and ligand-free structures. In the next step, we aligned the structures with DeepAlign [35] and calculated tunnels in each pair of structures. Finally, we analysed the changes and differences to determine the properties of potentially relevant tunnels.

### Machine-learning predictor for pocket distinction

The main goal of this part of the pipeline was to create a predictor which would be able to assess and differentiate between buried and surface-exposed protein pockets. For the training of the predictor, we manually labelled 200 pockets. We analyzed the distribution of the Enzyme Commission (EC) classes in the dataset and randomly collected samples in quantities that matched the EC class distribution. Features were extracted from Fpocket 2 output, and an additional “Exposed ratio” feature was included, representing the number of solvent-accessible residues. In total, 20 features were used (Table S1). Pockets were categorized into three classes: buried, borderline, and surface, based on manual inspection. The following software was used for the training of the predictor: Python 3.9.7, NumPy 1.26.2, Pandas 1.4.3, Scikit-learn 1.1.1. We tested the Support Vector Machine (SVM), K-Nearest Neighbour (KNN), Shallow Neural Network (ANN), Gaussian Naive Bayes, and Random Forest as classifiers. In each case, we applied a grid search with five-fold cross-validation for tuning hyperparameters of the algorithms (Table S2) and conducted data preprocessing, including Kolmogorov–Smirnov feature filtering [36]. The performance was evaluated using accuracy, precision, recall, FPR, and F1 measures because the dataset was balanced. For validation, we employed an independent test set of additional 100 manually labelled samples, mirroring the class distribution of the training set (Table S3). The best predictor was then used to classify all calculated pockets.

### CaverDock energy analysis

CaverDock 1.1 [37] was used to analyse the ligand pathways in all cases in the dataset with successfully calculated tunnels. CaverDock is a tool designed for rapid analysis of ligand transport. It enables fast simulation of the binding and unbinding of ligand molecules through protein tunnels. CaverDock achieves short calculation times which makes it well-suited for virtual screening applications. The current version of CaverDock uses CAVER 3.02 for the pathway identification and AutoDock Vina 1.1.2 as the docking engine, applying its docking algorithm and empirical scoring function without any modifications. Each CaverDock calculation requires the receptor, ligand, and tunnel input files and the configuration. The tunnel is discretized into a set of discs which are used to guide the ligand through the protein during the simulation. To produce the trajectories for the study, we used the lower-bound CaverDock calculations. In each step of the CaverDock lower-bound trajectories, the ligand is constrained to a disc, and the docking algorithm docks the molecule to the disc and optimises the conformation. Apart from the selected drag atom which is constrained to the disc (Table S4), the rest of the molecule can move freely. Then the ligand is moved to the next disc and the process is repeated until the molecule reaches the end of the tunnel. The outputs are the ligand trajectory and the energetic profile of the un/binding process.

The information from the relevant cognate KEGG [32] reaction was used to collect the cognate ligand and to set the drag atom used to guide the molecule during the simulation by processing the information with Reaction Decoder Tool [38] and RDKit (https://github.com/rdkit/rdkit)*.* The processed ligand and enzyme structure files were then converted to PDBQT using the scripts from MGLtools 1.5.6 [39]. The tunnel 3D representations in PDB format were discretized into a set of discs using the Discretizer tool from the CaverDock package. Finally, the grid box around the tunnel and the configuration file were prepared by the prepare-config script from the CaverDock package. The direction of the simulation was defined based on the type of the ligand, binding for substrates and unbinding for products. Only the lower-bound trajectory was calculated and analysed. Important energy values were extracted from the energy profiles manually for the validation dataset and automatically in the annotation pipeline: E_Bound_, E_Max_, and E_Surface_. The energy barriers were calculated as E_a_ = E_Max_ − E_Bound_ for the products and E_a_ = E_Max_ − E_Surface_ for the reactants.

The CaverDock tool has been tested extensively and used on various datasets in previous publications [20, 21]. However, validation of the quality of predicted trajectories from CaverDock has not been done by any method approaches based on Molecular Dynamics (MD). We validated CaverDock by running classical MD simulations and Adaptive Steered Molecular Dynamics (ASMD) [40]. In contrast with unbiased MD, the ASMD method applies constant external force on two atoms in the simulated systems. This can be used to simulate unbinding or binding ligands through tunnels. The direction of the movement is set by selecting the steering atoms to move the ligand in the direction of a selected tunnel by lengthening or shortening the distance for unbinding or binding respectively. While changing the distance between those two atoms, the ligand moves in the given direction, but it can follow the curves of the tunnel which allows it to move through the protein. The steering atoms or the direction are not changed during the simulation. In ASMD, the simulation is divided into multiple stages. During each stage, the steered simulation is performed in several parallel replicas, and the Jarzynski average [41] is calculated at the end of that stage. The simulation then proceeds by selecting the single trajectory with a work value closest to the Jarzynski average. The next stage continues from the selected trajectory. The Potential of Mean Force (PMF) is calculated at each stage, and at the end of the ASMD simulation, the segments of the PMF are combined to form the complete PMF. For the validation, we selected eight cases from the dataset with protein structures which had 2–4 well-defined tunnels and the cognate product bound inside (Table S4). To prepare the complexes for the validation unbinding simulations, we selected the lowest-energy binding pose from the CaverDock analysis of the first tunnel, extracted the pose, and saved it in the protein structure. The complexes were then processed by several tools, minimised, and equilibrated before running the MD simulations with AMBER 16 [42–51].

Before we started with the biased unbinding simulations, we ran classical MD simulations of *System #3* and *System #4* (Table 2) to showcase the need for biased MD simulations [15, 52] and approximative methods for the study of ligand unbinding [19]. We used the prepared complexes and ran 3 replicas of 1 µs simulations to study the behaviour of the complexes and the potential unbinding of the ligand molecules. Next, the unbinding trajectories were calculated with ASMD. The following parameters were used: 25 parallel simulations, 2 Å stages, a velocity of 10 Å/ns, and a force of 7.2 N. The protein atom for the steering was different for each tunnel. The ligand atom for steering was selected as the one closest to the centroid of the molecule. Lastly, we ran MD simulations with ligand-free structures to generate ensembles of protein snapshots to study how much CaverDock results change when using dynamic structures. We used the same settings for the preparation of the systems, minimisation, and equilibration. We ran 50 ns of production MD, saved 25,000 snapshots, and from these we collected 100 snapshots covering the entire MD simulation. We calculated the tunnels in selected snapshots using CAVER and the transport of ligands through the snapshots with CaverDock. Then, we collected and averaged the energy values for each snapshot and tunnel in every system. Finally, the Potential of Mean Force profiles from ASMD and CaverDock energy profiles from a single static structure and averaged values were compared. We qualitatively analyzed the results by comparing the order of the calculated profiles based on their maximum energy for each tunnel and the number of matching profiles between the two methods (e.g. if the profile for a tunnel is the first one by ASMD and in CaverDock it is considered as a match). We are aware that both MDs and CaverDock use different methods for both parametrisation and evaluation of the transport energy. Our main aim was the qualitative comparison to see if the molecules could unbind through the selected tunnels.

**Table 2:** The comparison of Potential of Mean Force profiles obtained from ASMD simulations and energy profiles from single structure or averaged CaverDock calculations over snapshots from MD simulations

Case	Enzyme	Ligand	Number of tunnels	Match with static CaverDock	Match with averaged CaverDock
System #1 (PDB ID 1OTW)	Pyrroloquinoline–quinone synthase	Pyrrolo-quinoline quinone	3	1 out of 3	1 out of 3
System #2 (PDB ID 2BFN)	Haloalkane dehalogenase LinB	*trans*-3-Chloro-2-propene-1-ol	3	3 out of 3	3 out of 3
System #3 (PDB ID 2RFY)	Cellobiohydrolase	Cellobiose	3	0 out of 3	1 out of 3
System #4 (PDB ID 2UWH)	Cytochrome P450 BM3	11,14,15-Trihydroxyicosatrienoic acid	3	2 out of 3	3 out of 3
System #5 (PDB ID 4E2Z)	C-3′-methyltransferase	Se-adenosyl-l-selenohomocysteine	3	3 out of 3	3 out of 3
System #6 (PDB ID 5EDT)	Cytochrome P450 CYP121	(4*S*)-4-(5,5-Dimethylcyclohex-1-en-1-yl) cyclohex-1-ene-1-carboxylate	4	0 out of 4	0 out of 4
System #7 (PDB ID 3ORW)	Phosphotriesterase	*N*-(6-Aminohexanoyl)-6-aminohexanoate	2	2 out of 2	2 out of 2
System #8 (PDB ID 5U6M)	UDP-glucosyltransferase	Uridine 5′-diphosphate	3	3 out of 3	3 out of 3

## Results

### Automatic annotation

#### Annotation of the filtered PROCOGNATE dataset

The summary of the filtering of the PROCOGNATE dataset is given in Table 1. Out of the 17,092 unique PDBs, the ligand was not present in the biological unit in 193 cases, so we had to use the asymmetric unit instead. In 133 cases, the ligand was not present in the PDB at all—when three-letter codes for ligands did not match the bound ligand code in the dataset. In 1058 cases, the ligand was not inside any of the calculated pockets but rather at or near the protein surface, therefore it was impossible to define any pocket which contained the ligand. In 337 cases, there were errors in the pocket calculation and the tool failed to predict any pockets. We looked at the representation of the enzyme classes defined by their catalysed reaction and classification by the EC numbers in the 15,697 cases with successfully calculated pockets, and all EC classes were represented in the dataset: EC 1 (25.1%), EC 2 (38.5%), EC 3 (20.4%), EC 4 (7.7%), EC 5 (4.3%), EC 6 (3.6%) and EC 7 (0.4%). Concerning the tunnel detection, no tunnels were found in 526 cases, and 739 cases finished with errors. This could stem from the following: (i) the pocket was at the surface of the protein and the CAVER algorithm was unable to calculate any tunnels, (ii) the automatically set starting point was in an incorrect position, or (iii) the space was too narrow for the 0.9 Å probe during the calculation, and the tunnel calculation failed.

#### Validation of annotations

The twofold validation was used to evaluate the usability of the proposed pipeline. We analysed the collected annotations for residues essential for function, i.e., catalytic or binding residues, in selected binding pockets. By searching UniProt and CSA, we managed to find annotations for 11,046 protein structures, and for 4651 structures, we found no information on essential residues (Table 1). Out of 11,046 annotated cases, 76% matched the essential residues with the pocket-lining residues.

We further investigated the impact of the selection of the studied pocket on the performance of the pipeline. Using the ligand coverage, i.e., the fraction of the molecule overlapping with a pocket, we discriminated between three scenarios: (i) the ligand belonged only in one pocket (single pocket), (ii) a part of the ligand was found in another pocket, but the ligand was occupying the main pocket by 10% more than other pockets, (iii) the ligand occupied multiple pockets, and the difference was less than 10%. In the third scenario, e.g., when half of a ligand was inside one pocket, and the second part lay in another (Figure S1), we selected the pocket with the highest druggability score. To this end, we looked at how often the selected pocket has the best Fpocket and druggability scores in the matching/mismatching/no annotations subsets (Table S5). In these subsets, the selected ligand-binding pocket was top-ranked by Fpocket scores only in 43%, 27%, and 41% of the cases. In the case of druggability scores, it was 23%, 12%, and 17%, respectively. These values were surprisingly low, implying that selecting the pocket based on calculated scores would lead to a high number of errors. On the other hand, based on the 75% overlap of the selected pockets with annotated essential residues in structural databases, we can say that the approach of selecting the pocket based on the ligand location is significantly better than a blind selection of the best pockets ranked by Fpocket score or druggability. Furthermore, using the same settings for the Fpocket calculation for all proteins in the dataset seems insufficient as it led to cases where the ligand overlapped with multiple pockets. In addition, selecting the pocket by predicted scores is not generally applicable to any ligand-free structure without available essential residue annotations. A solution could be to extrapolate the location of the ligand and selected pocket from structurally similar proteins.

**Fig. 2:**
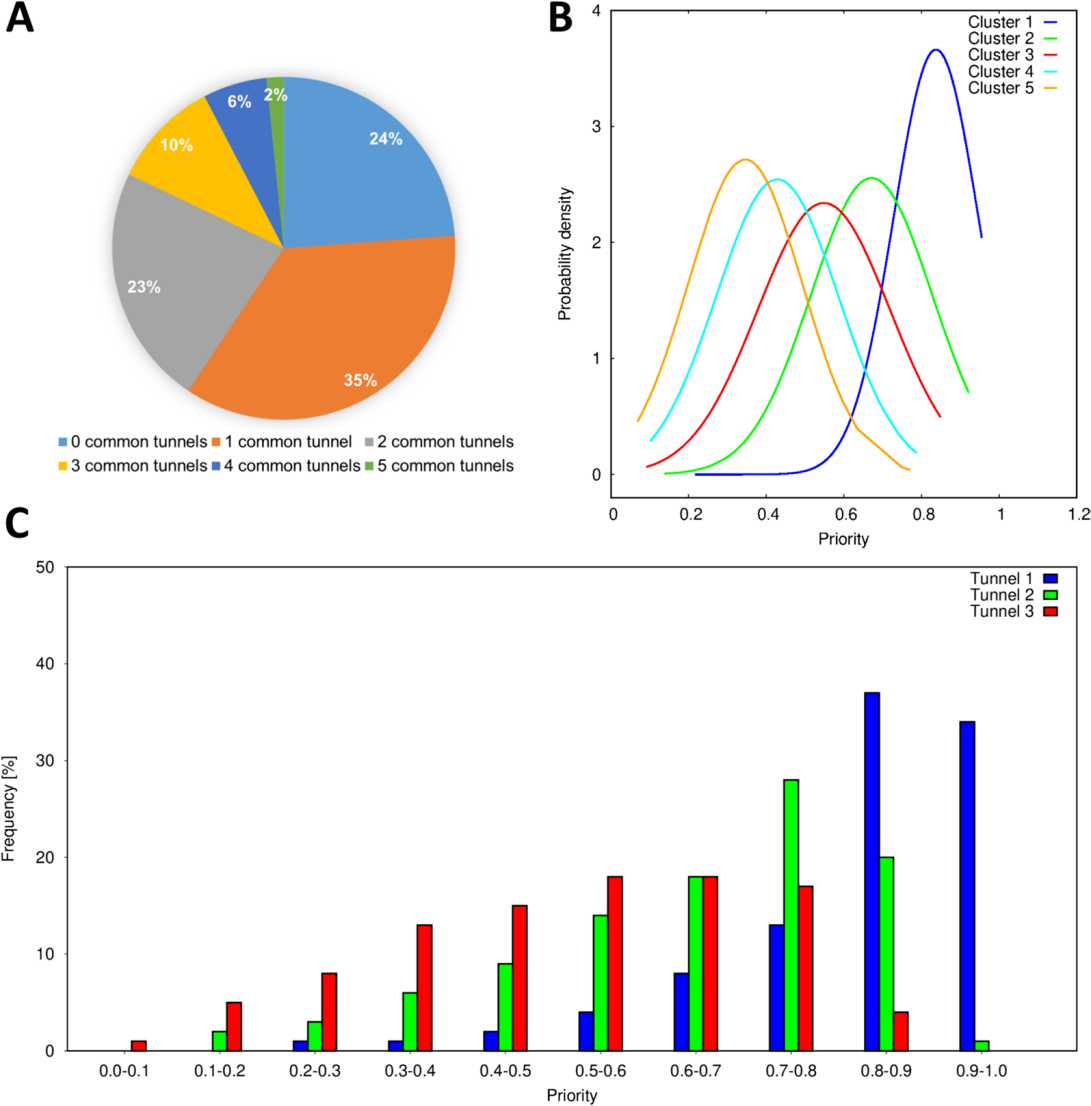
Analysis of tunnels in pairs of ligand-bound and ligand-free structures and the entire annotated dataset. **A** The number of common tunnels found in both ligand-bound and ligand-free structures. **B** Probability of distribution of CAVER priority score for the best five clusters in pairs of structures. **C** Distribution of the priority score for the first three tunnels in all proteins from the dataset with calculated tunnels. The analyses show that the first three tunnels are commonly present in enzyme–ligand complexes and ligand-free structures. These tunnels have the best geometrical parameters and are suitable for ligand un/binding. Tunnels with the priority above 0.55 could be potentially biologically relevant

In the second part of the validation, we analysed how the presence of a ligand impacted the geometry of tunnels in proteins in pairs of ligand-bound and ligand-free structures to analyse the potential effect of induced fit in the structures. We used the priority score in CAVER 3.02 to calculate how many out of the top 5 tunnels identified in ligand-bound structures could also be found in the top 5 tunnels of ligand-free structures (Fig. 2A). In the 2904 studied pairs, we found no common tunnels in 24% of the cases. This could be caused by the absence of the ligand in the structure, which led to a narrower binding site and impacted the geometry of calculated tunnels. In this category, no tunnels were found in the ligand-free structure in 146 cases, and only one tunnel, which did not match with any of the tunnels from ligand-bound structures, was found in 139 cases. In the rest of the structures, 35% had one common tunnel. Based on the results, we observed that it was generally rare for a protein to have more than three potentially biologically relevant tunnels. We collected the priority scores for each of the five ranks of common tunnels and calculated the probability distribution to further study the clusters and define a metric for potentially relevant tunnels (Fig. 2B). We concluded that the tunnels with the priority above 0.55, the average priority score of the third tunnel, could be potentially relevant, with geometrical properties suitable for ligand un/binding. We suggest that for screening purposes, users should focus only on the first three tunnels calculated by CAVER 3.02 or use more tunnels with a priority score above 0.55. This recommendation is aimed only at the cases in which there is no previous information about the relevancy of tunnels in the studied protein. Based on these findings, we focused only on the first three tunnels in our subsequent data analyses.

### Machine-learning predictor for pocket discrimination

Since tunnel calculations are of little use for the surface binding pockets in the annotation pipeline, we trained a machine-learning predictor for identification of such pockets. We used KNN, Random Forest, SVM, ANN, and Naïve Bayes to discriminate between buried and surface binding pockets. We tested two annotation strategies: a three-class problem (buried, borderline, surface) and a two-class problem in which the buried and borderline classes were merged into one (Table S6, Figure S2).

The Naïve Bayesian predictor was used as a simple baseline, and while it showed the highest value of 1-FPR of 90% and 93% on the training dataset for three- and two-class problems, respectively, it failed to identify any buried samples in the test dataset. For the three-class problem, the ANN achieved the highest accuracy (54%) and F1 score (50%), and the second-highest 1-FPR score (67%) on the test set. ANN was also among the top-performing models for the two-class prediction, with all three metrics of 70% on the test set. Despite featuring lower absolute values, the three-class prediction results were similar to those for a two-class predictor if the baseline accuracy of a completely random prediction was taken into account (33% vs. 50%). Therefore, we selected the ANN-based three-class predictor to annotate the successfully calculated pockets (Table 1).

To get a better understanding of our results, we conducted several additional analyses. Since KNN achieved the highest 1-FPR score on the three-class dataset and performed similarly to ANN on the two-class dataset, we further examined whether the misclassified cases differed between the two models. There was almost no overlap in misclassifications in the three-class dataset, except for a few cases (i.e., 8 buried pockets classified as surface) in the test set. Moreover, while both predictors showed low performance in borderline cases and similar performance in buried cases, the ANN predicted most surface pockets correctly. Furthermore, in addition to evaluating our predictors on the test set, we also constructed learning curves to determine whether expanding the training set (beyond 160 training data points in each fold) could enhance the performance of the predictors (Figure S3). However, the curves did not provide any evidence that the accuracy would increase if more data points were added for training. Finally, the feature pre-selection based on the two-sample Kolmogorov–Smirnov test did not improve the results, so we used the entire set of features in our final predictor. The python code for the pocket discrimination predictor is available at https://github.com/Faranehhad/Large-Scale-Pocket-Tunnel-Annotation.git and as a part of the supplementary material.

### CaverDock energy analysis

#### CaverDock annotation results

We analysed 14,432 proteins with calculated tunnels with CaverDock. We were not able to produce ligand trajectories for 1244 protein–ligand systems (Table 1) due to several factors: (i) we had problems with the automatic parsing of ligand data from KEGG, (ii) protein structures contained parts of DNA or RNA which caused the receptor preparation to fail, (iii) we failed to discretize the tunnels for CaverDock because they were extremely short, represented by only one dummy sphere or one sphere encompassed by another, or (iv) we discarded the cases in which the lower-bound CaverDock calculation did not finish within 48 h on 4 CPUs. Based on the tunnel priority distribution, we analysed the energies of ligand un/binding in up to three tunnels found in each protein. In 13,188 successfully calculated protein–ligand systems, we produced 29,752 energy profiles: 12,804 trajectories for the tunnel 1, 9465 for the tunnel 2, and 7483 for the tunnel 3.

#### MD simulations for validation of CaverDock trajectories

Both unbiased and biased MD simulations were used to validate the quality of CaverDock results. We simulated three replicas of 1 µs unbiased MD simulations for cellobiohydrolase with cellobiose and cytochrome P450 BM3 with 11,14,15-trihydroxyicosatrienoic acid (*System #3* and *#4* in Table 2, respectively). The ligand remained in the binding site, and we did not observe unbinding in any replicas. This result showed the importance of applying bias in MD to study events such as ligand unbinding. Furthermore, it demonstrated the applicability of approximative methods for the simulation of un/binding to save computational time and effort since unbinding was not observed even in these long simulations. We qualitatively compared the match between the Potential of Mean Force profiles (PMF) from ASMD and CaverDock calculations. We used the CaverDock trajectories from the single static structure and the averaged CaverDock results from 50 ns MD snapshots (Table 2). We show the highest energy value in the profile E_Max_ for the static and the averaged CaverDock calculations in Table S7.

**Fig. 3:**
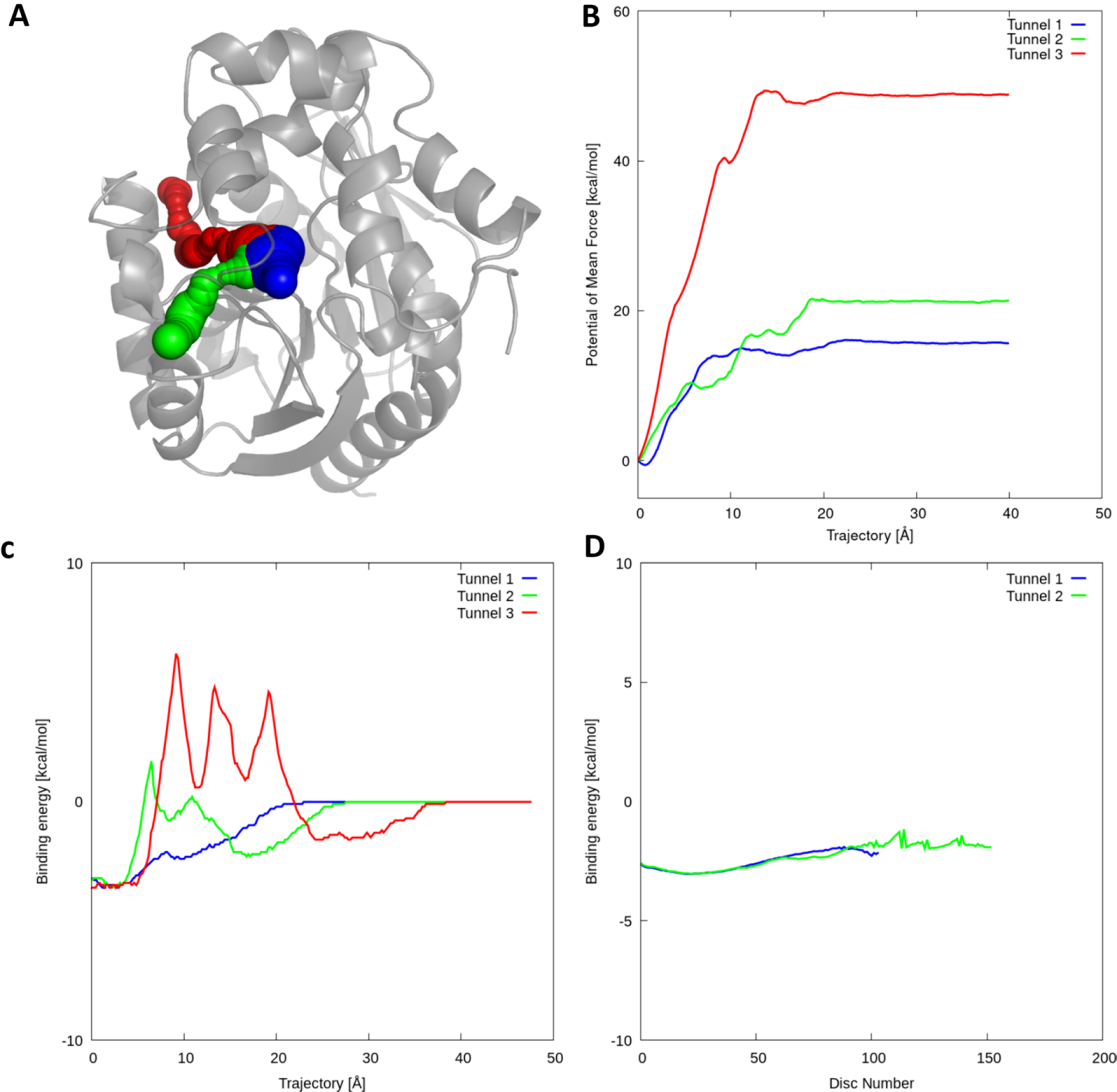
Results from CaverDock validation for haloalkane dehalogenase LinB with trans-3-chloro-2-propene-1-ol. **A** Visualisation of the protein structure (PDB ID 2BFN) with analysed tunnels showed as spheres: tunnel 1 (blue), tunnel 2 (green), tunnel 3 (red). **B** Potential of mean force profiles from ASMD simulations. **C** Energy profiles from static CaverDock calculations. **D** Averaged CaverDock energy profiles from 50 ns simulation snapshots. The third tunnel was not present in the MD snapshots. The System #2 showed qualitative agreement between the ASMD and CaverDock results

In the case of *System #1* (Figure S4), the energies for tunnels 1 and 2 were similar, but the order was swapped compared to the ASMD simulations. Both tunnels were not frequently open in the 100 snapshots (Table S7). Moreover, the priority of tunnel 1 was lower in MD snapshots, so both tunnels 1 and 2 seem to be feasible for ligand binding. The ligand was not able to unbind through tunnel 3 in ASMD simulations, which agrees with the large barrier found in CaverDock energy profiles. *System #2* had a match for all three tunnels (Fig. 3). In ASMD the ligand was able to unbind with difficulties in tunnel 3, but the force started to unfold the part of the protein that was used for steering the simulation. This result is in accord with the large CaverDock barriers. In *System #3*, there was no matches between ASMD and CaverDock results for the static structure (Figure S5). The use of averaged results from MD snapshots improved the results, as the energy profile for the tunnel 2 was the highest. We concluded that the loops around tunnel 2 made it too wide open in the static structure and biased the results. The ligand in *System #4* unbound successfully in both tunnels 1 and 2 (Figure S6). On the other hand, it did not unbind through tunnel 3 and remained stuck in the binding site. Therefore, we deduced that both tunnel 1 and 2 could be preferred by the ligand. In *System #5*, there was no unbinding observed in tunnels 2 and 3 (Figure S7). The results from all the simulations agreed. The inability to pass through the tunnels in ASMD was reflected in the barriers in both types of simulations. *System #6* had no matches between CaverDock and ASMD, and the use of averaged energies from snapshots did not improve the results (Figure S8). The crystal structure seemed too compact and presumably did not have enough time to open during the short MD simulation of the complex. In *System #7*, the ligand was able to unbind successfully through tunnel 1 but was not able to pass through tunnel 2 (Figure S9). CaverDock results agreed with ASMD, so we had a good match across all simulations. In the case of *System #8*, the ligand preferred tunnel 1 over tunnel 2 and was not able to pass through tunnel 3 (Figure S10). Static CaverDock showed similar energies for both tunnels 1 and 2, and the results were improved in MD snapshots, where we saw a slightly higher barrier in tunnel 2. It indicated that both tunnels 1 and 2 could be used by the ligand. Regarding this validation dataset, we observed that some profiles both from PMF and CaverDock were too high in comparison with the other profiles, e.g., *System #5* and *System #6*, suggesting the low probability of these tunnels being used for ligand transport. The RMSD values for 50 ns MD simulations without ligand and ASMD simulations with ligands are listed in Table S11.

### Data analysis

The ANN predictor was used to discriminate the pockets based on their type for all cases within the dataset. In the case of the pockets for which we did not manage to calculate tunnels, 508 pockets were predicted as buried, 160 as borderline, and 597 as surface. This was a surprising finding since we expected all these pockets to be predicted as surface pockets. In the second part of the dataset, i.e., the cases with pockets and successfully calculated tunnels, 3552 cases were predicted as buried pockets, 3178 as borderline, and 7702 as surface (Table 1). In the subset of proteins for which we were able to identify pockets and tunnels, we coupled the predictions with the information about tunnels (Table S8). We binned tunnels similarly as in the study of Pravda et al*.* [13]: short tunnels under 5 Å, medium-length tunnels between 5 and 15 Å, and long tunnels over 15 Å. In tunnel 1, we see a significant overlap between the categories of pockets with corresponding tunnel lengths. The vast majority of tunnels (75%) in buried cases were either medium or long. Borderline cases were defined as a separate category because during manual annotation, it was difficult to assess if pockets were completely open on the surface or partially buried. For this category, we had 41% short, 49% medium, and 10% long tunnels. For the surface cases, 74% were short tunnels. Thus, our predictor proved successful in its predictions for tunnel 1 and could be a useful tool for assessing whether the calculation of tunnels in a protein makes sense or there is just a surface cavity. We carried out a similar analysis for tunnels 2 and 3, but these tunnels were of lower priority and always longer than tunnel 1. Therefore, almost all the tunnels were either medium or long. Based on this result, we defined tunnel 1 as the only reliable descriptor of the relationship between the predicted pocket type and tunnel length. Moreover, the proteins with tunnels shorter than 5 Å could potentially be discarded since they were calculated for pockets predicted as surface pockets and were, therefore, irrelevant to the tunnel analysis. The main benefit of the predictor is the possibility of pocket annotation in enzyme structures with very narrow tunnels, which would not be found unless the user used a smaller probe during the calculation, or when the tunnel calculation fails. One could also use the predictions to decide whether calculating and analysing tunnels is worthwhile for a particular protein structure. Since the predictor does not require the presence of a ligand in the structure, it is also generally applicable for ligand-free structures.

We studied the geometry of the first three tunnels in more detail. The distribution of tunnel priority scores for all cases with calculated tunnels is presented in Fig. 2C. Importantly, we observed the same trend in the priority scores as in the analysis of pairs of complex and ligand-free structures. The throughput of tunnels 2 and 3 was lower because they were narrower, longer, and more curved than the tunnel 1 (Figure S11). This is not surprising since the priority score is related to the geometrical tunnel properties. Therefore, the priority score should be a sufficient metric for screening purposes. We continued this analysis by separating the dataset based on EC numbers (Figure S12). Tunnels were present in proteins from all EC classes, which was in agreement with previous studies [13]. The tunnel priority followed the same trend in all the classes apart from EC 7 due to the low number of cases in the dataset. We did not observe any major differences in the geometrical properties, which would otherwise indicate that certain EC classes preferred tunnels with specific geometries. We also studied the number of tunnels in each EC class with a priority higher than 0.55 (defined in the analysis of pairs of structures). Apart from EC 7, the results were similar for all EC classes (Figure S13). For future tunnel analyses, it might be worthwhile to compare subclasses to see more significant differences in tunnel geometries.

**Fig. 4:**
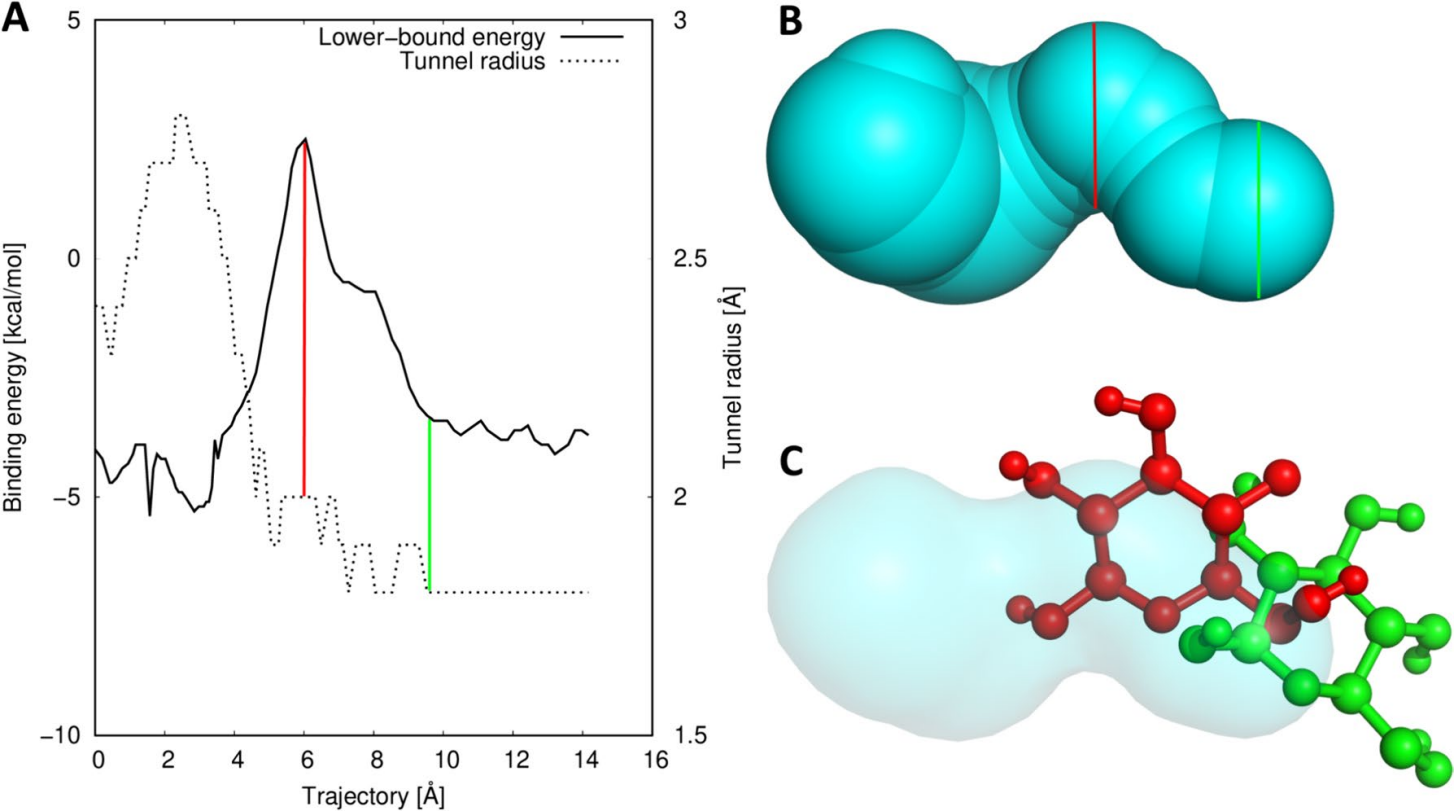
An example of the case with a large difference between the energetical maximum identified by CaverDock and the geometrical bottleneck identified by CAVER. **A** Energy profile from CaverDock (solid) and the geometric profile from CAVER (dotted). The tunnel region with the energy maximum is highlighted with the red line, and the region with a geometric bottleneck is highlighted with the green line. **B** Visualisation of the tunnel with highlights corresponding to the energy profile. **C** Visualisation of the cognate ligand β-d-glucose conformations extracted from the trajectory from tunnel 1 of the structure of glucose dehydrogenase (PDB ID 2VWG). The binding pose based on the energy maximum (red) and geometric tunnel bottleneck (green)

Next, we studied whether the geometrical bottleneck, i.e., the narrowest part of a tunnel, was the best hot spot for mutagenesis to improve ligand binding and selectivity. For this purpose, we collected the maximum energy E_Max_ from each CaverDock trajectory. In the next step, we compared the location of the energy maximum and the geometrical bottleneck in the tunnel (Fig. 4). We tracked how often the maximum energy was in the disc with the lowest radius or in its vicinity (1.5 Å, 3 Å, and 5 Å). The match between the energy and geometry bottleneck was around 50% for the exact disc and 75% for the 5 Å vicinity (Table S9). The mismatch showed that studying the geometry of the tunnel is a good starting point for quantifying the likelihood of a tunnel being used for ligand transport. Furthermore, the analysis of the energy profiles by approximative methods can be the source of valuable information and help with the identification of other important hot spots for the study and the modification of the ligand transport. The analysis was run with cognate ligands; therefore, these molecules should be recognizable by the enzymes. The results might change for a set of ligands of a larger size or with physicochemical properties different from the cognate ligands.

CaverDock energy profiles were used to analyse the ligand preference of tunnels based on the energy barriers. We compared the maximum energies in up to the three tunnels and selected the best one. The first third of the profiles was removed in order not to include peaks of energy at the beginning of the profiles caused by clashes at the bottom of the tunnel. In the 13,158 proteins with successful CaverDock calculations, tunnel 1 had the most favourable energy in 75% of the cases and tunnel 2 in another 19% of the cases (Table S10). Therefore, for screening purposes, the analysis of tunnel 1 (or at most tunnel 2) would be enough for more than 93% of proteins. Based on these results, tunnel 1 had the best properties for ligand un/binding and would be the most biochemically relevant.

Finally, we studied how well the cognate ligands were recognised by their receptors. We analysed the distribution of energy maxima (Figure S14) and the energy barrier (Figure S15). In the case of tunnel 1, which was the most preferred tunnel for ligand un/binding, almost 80% of the E_Max_ values were in the range between − 10 kcal/mol to 5 kcal/mol, and the energy barriers E_a_ were in the range between 0 kcal/mol and 10 kcal/mol. Both values were highly correlated for cognate ligands, and the Pearson's correlation coefficient was 0.98 for energies from all three tunnels. Using E_Max_ seems to be equivalent to E_a_ for cognate ligands and probably for other natural substrates, which should be transported reasonably fast and bound in the active site. For inhibitors, both values could have different meanings as the molecule does not need to pass the entire way to the active site, but the binding affinity must be much stronger. In such cases, we recommend using E_Max_ values as they are easier to collect and interpret. We analysed the data split by pocket classes, EC numbers, and cognate ligand similarity. Still, all the datasets showed similar trends without major differences (data not shown), implying that ligand trajectories are case-specific rather than showing some general trends in different groups of enzymes.

## Discussion and conclusions

We describe the development of an automatic pipeline for the analysis of pockets and tunnels in enzymes and its application to study enzyme–cognate ligand complexes. The results provided a way to select potentially biologically relevant tunnels. The proposed approach can be used for extending large protein datasets for structural analyses and screenings. We analysed more than 17,000 cognate enzyme–ligand complexes. We were able to successfully annotate and analyse structural features and the energetics of ligand passage through tunnels in 13,158 enzyme structures. The tunnel data collected in this study has been made publicly available as part of the ChannelsDB 2.0 database [14]. Each part of the pipeline was thoroughly validated, and the data showed that binding pockets selected based on the location of a bound ligand had a good overlap with catalytic and binding residue annotations from the structural databases. Therefore, bound ligands can be used to extend the datasets for pocket and tunnel analyses. Our experiments showed that selecting the pocket purely by score or druggability from Fpocket would be significantly less precise. On the other hand, our pipeline is limited to enzyme structures with bound ligands, which limits its use. However, this limitation is merely a consequence of being able to classify enzymes and their cognate ligands based on their reactions, which are available in public databases. Extrapolation of ligand positions among homologous protein structures could remove this limitation for many structurally or functionally related proteins. Furthermore, the use of the pipeline to detect non-cognate ligands would probably provide less precise results as it would be harder to select the correct pocket for the following analyses and calculations. Due to the development of AlphFold [53] and AlphaFill [54] the protein engineering community has access to a staggering amount of new protein models and modelled complexes. As an example of the adaptability of our pipeline, we contributed to the update of ChannelsDB 2.0 database [14]. We calculated tunnels for a dataset based on protein structures from AlphaFill with known cofactors. The position of cofactors was used to define the binding pocket and for the later calculation of tunnels in the model structures.

The presented machine learning predictor for the annotation of pockets has proven to be efficient in deciding on the type of pocket. Based on the test set, the machine learning predictor demonstrated the accuracy of 54% and 1-FPR metric of 75% of buried pockets in the three-class prediction. While there still is room for improvement, the current version shows reasonable performance for selecting whether a particular enzyme and pocket are viable for tunnel calculations. Most importantly, it uses the readily available features from the Fpocket, making it easy to obtain these necessary features. At the same time, we release the training data together with the scripts to encourage follow-up studies to improve the predictor, e.g., by considering other, more discriminative features. The structural analyses revealed that it is possible to select potentially biologically relevant tunnels both in ligand-bound and ligand-free structures. Tunnels are present in the enzymes of all seven EC classes. Strikingly, the ligand transport calculations revealed that the energetic maximum was not in the geometrical bottleneck in 50% of analysed tunnels. Therefore, energy profiling provides a highly relevant information about hot spots for enzyme engineering. The comparison of CaverDock energetic maxima for calculated tunnels in each enzyme structure indicated that tunnel 1 had the lowest energy barrier in 75% of cases. This shows, that the energy analysis by CaverDock is valuable addition to the study of tunnel geometry when multiple tunnels can be relevant for a specific ligand. To improve the predictive power of such analysis, the study of geometrical and energetical bottlenecks should be done on a large set of dynamic snapshots. The results from a single structure may be biased by the enzyme conformation in the crystal structure.

The knowledge and data acquired in this study will be important for future screening studies and the development of computational tools. We showed that the presented pipeline could be used to generate features for machine learning predictors and to provide valuable information for key repositories of biological data, such as PDBe Knowledgebase [55]. The validation of CaverDock against MD simulations proved that approximative methods are precise enough for fast energetical analyses of ligand passages. Approximative methods and enhanced sampling simulations are necessary to simulate ligand transport within reasonable times. Thus, we recommend energy calculations with approximative methods for protein engineering studies. Our comprehensive analysis of protein tunnels and the passages of cognate ligands let us formulate the following recommendations for the protein engineering community:

1. For analysis of tunnels in enzymes, start with the literature search and exploration of databases to determine essential residues, identify the location of the binding pocket, and discover transport pathways, whenever possible.
2. The pocket(s) that contains the essential functional residues should be preferred. In the systems with unknown essential residues, the pocket which contains a bound cognate ligand of the enzyme should be used. If there are no ligand-bound structures for the enzyme of interest, analyse available structures of homologous enzymes which contain the ligand. We recommend caution when selecting the binding pocket based solely on the predicted scores by the tools for pocket calculation.
3. The most important step of the tunnel analysis is to set the starting point correctly. When annotations of essential residues are not available the conserved residues are another possibility. Otherwise, we recommend using the residue inside of the selected pocket, closest to the centre of the biological unit or the analysed protein chain in the asymmetrical unit to start the tunnel calculation from the deep part of the pocket. An incorrectly set starting point may hinder the tunnel calculation and impact the geometry of found tunnels.
4. Selection of the biochemically relevant tunnel(s) should be preferably made based on the experimental literature data. When no such information is available, either focus on the first tunnel in a screening scenario, or the first three tunnels according to the highest priority score. CAVER users are advised to inspect the tunnels with priority scores above 0.55. If none of the found tunnels has a priority score above this value, select a different starting point and redo the calculations.
5. If the starting point for tunnel calculation is selected correctly and the first tunnel is shorter than 5 Å, the binding pocket could be located on the surface and tunnel analysis might not be relevant.
6. Analysis of tunnels should be complemented by the study of substrate or product passage whenever possible.
7. Use the ranges of energy barriers defined in this study to filter out molecules with poor (un)binding (E_Max_: − 10 kcal/mol to 5 kcal/mol, E_a_: 0 kcal/mol to 10 kcal/mol) for energetic analyses of ligand passage by the approximative method CaverDock [16]. Other methods available for this purpose are SLITHER [56], MoMA-LigPath [57], GPathFinder [17], and ART-RRT [18].
8. Binding and unbinding studies by the approximative methods can be significantly enhanced by the analysis of an ensemble of structures obtained even from a short molecular dynamics simulations.

## Supplementary Information

Supplementary Material 1. Detailed description of the methods with settings and parameters; list of features and hyperparameters for the predictor; predictor learning curves; setup of ASMD simulations; detailed results from validations; details from structural and energetical analyses; tunnel parameters and presence in EC classes (PDF); filtered input dataset (CSV); information for 8 validation systems (CSV); list of PDB ID pairs of the complexes and ligand free structures (CSV); training dataset (CSV); testing dataset (CSV); predictor Python code (PY); training and testing dataset with labels from predictors (CSV).

## Data Availability

Supplementary materials contain: detailed description of the methods with settings and parameters, list of features and hyperparameters for the predictor; predictor learning curves; setup of ASMD simulations; detailed results from validations; details from structural and energetical analyses; tunnel parameters and presence in EC classes (PDF); filtered input dataset (CSV); information for 8 validation systems (CSV); list of PDB ID pairs of the complexes and ligand free structures (CSV); training dataset (CSV); testing dataset (CSV); predictor Python code (PY); training and testing dataset with labels from predictors (CSV). The python code for the pocket discrimination predictor is also available at https://github.com/Faranehhad/Large-Scale-Pocket-Tunnel-Annotation.
