# Supplementary material for: Large-scale annotation of biochemically relevant pockets and tunnels in cognate enzyme–ligand complexes
Source: J Cheminform. 2024 Oct 15;16:114. doi: 10.1186/s13321-024-00907-z (PMC11481355; doi:10.1186/s13321-024-00907-z)
Supplement: Supplementary file 1 — Supplementary Material 1. Detailed description of the methods with settings and parameters; list of features and hyperparameters for the predictor; predictor learning curves; setup of ASMD simulations; detailed results from validations; details from structural and energetical analyses; tunnel parameters and presence in EC classes (PDF); filtered input dataset (CSV); information for 8 validation systems (CSV); list of PDB ID pairs of the complexes and ligand free structures (CSV); training dataset (CSV); testing dataset (CSV); predictor Python code (PY); training and testing dataset with labels from predictors (CSV). [file 13321_2024_907_MOESM1_ESM.pdf]

Large-scale Annotation of Biochemically Relevant Pockets and Tunnels in Cognate Enzyme-Ligand Complexes

Vavra, O.[1,2], Tyzack, J.[3], Haddadi, F. [1,2], Stourac, J. [1,2], Damborsky, J. [1,2], Mazurenko, S. [1,2]*, Thornton, J.[3]*, Bednar, D. [1,2]*

[1] Loschmidt Laboratories, Department of Experimental Biology and RECETOX, Faculty of Science, Masaryk University, Kamenice 5, 625 00 Brno, Czech Republic; [2] International Clinical Research Center, St. Anne's University Hospital Brno, Pekařská 53, 656 91 Brno, Czech Republic; [3] European Molecular Biology Laboratory, European Bioinformatics Institute (EMBL-EBI), Wellcome Trust GenomeCampus, CB10 1SD Cambridge, United Kingdom

* Corresponding authors: S. Mazurenko and D. Bednar, Loschmidt Laboratories, Department of Experimental Biology and RECETOX, Faculty of Science, Masaryk University, Kamenice 5/A13, 625 00 Brno, Czech Republic; J. Thornton, European Molecular Biology Laboratory, European Bioinformatics Institute (EMBL-EBI), Wellcome Trust Genome Campus, CB10 1SD Cambridge, United Kingdom

E-mail addresses: mazurenko@mail.muni.cz (S. Mazurenko), thornton@ebi.ac.uk (J. Thornton), 222755@mail.muni.cz (D. Bednar)

Keywords: bottleneck, cognate ligand, cavity, enzyme, tunnel, machine learning, pocket, transport

SUPPLEMENTARY INFORMATION

Supplementary methods

1. Automatic annotation

1.1 Input data and annotation pipeline

The study uses data collected from the publication by Tyzack *et al.* [1] from 2018 and updated in 2021 for the purpose of our study. The data consists of enzyme-ligand complexes ranked by the similarity of the bound ligand with the cognate ligand from the KEGG [2] database calculated by the PARITY algorithm [1]. The following information is available for each case: KEGG Reaction number, EC Number, Protein PDB ID, Type of ligand (reactant or product), Bound ligand PDB ID, Cognate ligand KEGG number, and Similarity score (between bound and cognate ligands). In this analysis, we extracted all protein-ligand pairs with the Similarity score above 0.6, resulting in 35,882 entries. For

annotation purposes, we only used unique PDB IDs, which pruned the dataset to 17,092 cases (Table 1).

The pipeline starts with running the annotation module of HotSpot Wizard 3.0 [3] to search Swiss-Prot, UniProtKB [4], and CSA [5] for residue annotations using the sequence from the input PDB ID as a query. Moreover, the HotSpot Wizard module calculates solvent accessibility for each residue using the Shrake and Rupley algorithm [6] with BioJava [7]. The first assembly containing the biological unit is downloaded from PDBe [8] in mmCIF format. The list of known cofactor three-letter codes is assembled based on the list of cofactors from Fpocket 2 [9] and CoFactor Database [10]. Subsequently, the structure is cleared of any heteroatoms that do not belong to cofactors or bound ligands. The CIF file is then converted to PDB using the script cif2pdb from *gist.github.com/sbliven*. Each object from the original CIF is saved with a unique chain and matched by coordinates with the chains in the original PDB. From this point forward, only the PDB containing the biological unit is used in the rest of the pipeline, and only the first protein chain containing the bound ligand is analysed. When the biological unit is not available in PDBe, only the PDB within the asymmetric unit is used.

Next, pockets in the protein PDB are calculated using Fpocket 2 [9] with the following parameters: -m 2.8 (minimum radius an alpha sphere might have in a binding pocket) -n 10 (number of alpha spheres a pocket has to have close to alpha spheres of another pocket in order to be clustered together) -r 4.5 (parameter influencing the clustering of small pockets to larger pockets) -s 2.5 (parameter for multiple linkage clustering), and the list of the pockets related to the analysed protein chain is saved. In the next step, the information on whether these pockets contain the bound ligand is analysed with a simple in-house script. The script uses dummy spherical atoms from the files containing the 3D representation of each pocket. Then the atom coordinates of the bound ligand are checked to determine whether they are inside the dummy spheres. The information about how large a portion of the ligand is present in each pocket is collected and evaluated. In the situation where only one pocket is found to contain the ligand, this pocket is saved as the binding pocket. If multiple pockets contain the ligand (depending on the size and position of the ligand and pockets), the pocket with the largest portion of the ligand is selected if it contains at least 10 % more ligand atoms than the other pockets. In the case where there is less than a 10 % difference (for example, half of the ligand is in one, the other half in the second pocket), the pocket with a higher druggability score from Fpocket is selected. Then the pocket residues are checked if they were annotated by the HotSpot Wizard module in the previous step. This step is done for validation purposes.

Finally, the selected pocket is used for setting up the starting point for tunnel calculation by CAVER 3.02 [11]. First, the geometrical centre of the protein biological unit is calculated, and based on the distance to Cα atoms of each pocket residue, the closest pocket residue is selected. Next, the geometrical centre of the pocket is calculated, and in the previously selected residue, the nearest side-

chain atom closest to the pocket centre is found. After that, the coordinates of this atom are shifted by 0.5 Å in the direction of the vector connecting the atom and the centre of the pocket. After saving the coordinates for the tunnel starting point, CAVER is initialised with a probe radius of 0.9 Å and default remaining settings. When the tunnel calculation finishes, the results are collected and evaluated. The first five tunnels are saved based on the priority score from CAVER.

1.2 Validation of Annotations

We validated the output from the pipeline in two ways. First, we analysed whether the residue annotations from UniProt and CSA were available for the selected binding pockets. We analysed the cases with available annotations from the databases and calculated the overlap to see how precise we were in selecting the correct binding pocket. Then we continued with the analysis of pockets categorised by the ligand coverage scenarios. We looked at how often the selected pocket had the best Fpocket and druggability scores in the matching/mismatching/no annotations subsets. This validation would show if the pipeline is universally usable for ligand-free structures.

Second, we studied the impact of the ligand present in the protein structure on the tunnel quality and geometry to evaluate the applicability of the pipeline for proteins without residue annotations or proteins without bound ligands. We used the REST API in PDBe [8] and RCSB [12] to search for identical protein structures not containing any bound ligands apart from water molecules, using 100% identity and the E-value cut-off of $1e^{-15}$. Only the PDB-IDs of proteins with successfully calculated tunnels during the annotations were used for the search. In the cases where we found multiple empty structures for matching enzyme-ligand complex from the dataset, we picked the first PDB in the list. We downloaded the biological units for the ligand-free structures and aligned them to the complexes with DeepAlign [13]. Then we utilised the two structures as snapshots of one system and calculated the tunnels with the same settings as in the annotation pipeline. Using this workflow, we collected and analysed 2,904 pairs of protein-ligand complexes and ligand-free structures. To assess the structure pairs, we looked at the number of tunnels found in each pair and the tunnel properties provided by CAVER 3.02. We used the priority score from CAVER 3.02 that ranks the tunnels calculated by the tool. The priority score is calculated as a sum of the throughputs of all pathways in a given cluster, divided by the total number of snapshots that were analysed. In our case, the cluster of tunnels means that the same common tunnel was found in both structures in the pair. The throughput is calculated based on the cost function that takes into account the width and length of the tunnel, and it takes a value between 0 and 1, equal to the probability that the pathway is used as a route for the transportation of the substances [11].

2. Machine-learning predictor for pocket distinction

We initially used a simple metric of the ratio between buried and solvent-exposed residues in the binding pocket to assess its relevancy for tunnel calculation. However, preliminary data analysis

showed that this approach was not sufficient. Since none of the available pocket calculation tools offers any metric describing the pocket being buried or exposed on the surface, we developed a new predictor to discriminate between the two.

We manually labelled 200 proteins with calculated pockets from the dataset for training. We analysed the distribution of EC classes in the dataset and randomly collected the number of proteins from each EC class following the distribution. The features for training the predictor were collected from the output of Fpocket 2 for the selected pockets, calculated in the annotation pipeline. These features represent the accessible surface area, polarity, volume, hydrophobicity, and other properties of pockets. Moreover, we kept the Exposed ratio as an extra feature. This ratio is equal to the number of exposed solvent-accessible residues as a fraction of the total number of pocket residues. The total number of features used was 20 (Table S1). We labelled each pocket by three classes: -1 for buried samples, 0 for borderline samples, and 1 for surface samples, according to manual visual inspection of pockets and protein structures. The borderline case was introduced primarily due to occasional difficulty assigning labels to some binding pockets. We also considered combining 0 and -1 classes into one to check if this would improve the results. We realise that manual annotation of accessibility is unreliable, but it was impossible to generate the labels with other computational tools. The following software was usde for the training of the predictor: Python 3.9.7, NumPy 1.26.2, Pandas 1.4.3, Scikit-learn 1.1.1.

We used the Support Vector Machine (SVM), K-Nearest Neighbor (KNN), Shallow Neural Network (ANN), Gaussian Naive Bayes, and Random Forest classifiers. We applied GridSearchCV with 5-fold cross-validation for tuning hyperparameters of the algorithms from Scikit-learn: the function loops through predefined hyperparameters and fits the model to the training set to select the best parameters from a list based on the cross-validation error. The tuned hyperparameters and their corresponding ranges are listed in Table S2. For pre-processing data, we also tried a two-sample Kolmogorov-Smirnov test[14]. We used the Python SciPy function ks_2samp and the threshold of 0.11 to remove features scoring below the threshold from the dataset. Based on our results, we removed Apolar SASA, Volume, Alpha sphere density, and Alpha Sphere max dist for the two-class dataset. We tried three scenarios: (i) keep all three classes and features and tune the hyperparameters, (ii) absorb the borderline cases in the Buried class, remove the features based on the Kolmogorov–Smirnov test, and tune the hyperparameters, and (iii) the second scenario without the feature removal. We used accuracy, precision, recall, and F1 measure to report the performance of our predictor because the dataset was balanced:

Accuracy is defined as the percentage of correct predictions for the test data:

$$\text{Accuracy} = \text{correct predictions} / \text{all predictions}$$

F1 measure is calculated from the precision and recall:

$$\text{F1 measure} = 2 * (\text{Precision} * \text{Recall}) / (\text{Precision} + \text{Recall})$$

FPR is a false positive rate, which for a two-class problem is given as follows:

$$\text{FPR} = \text{false positives} / (\text{false positives} + \text{true negatives})$$

For the three-class problem, the most critical errors for our application are the cases when a pocket was assigned the label "1" (=surface) while the actual label is "-1" (=buried), as this will lead to falsely skipping the tunnel calculation step. Therefore, we used the following FPR formula for this case:

$$\text{FPR} = \frac{\text{Total number of buried pockets that are predicted as surface pockets}}{\text{Total number of buried pockets}}$$

To establish the statistical confidence of our evaluations, we ran the code 100 times with different random seeds and reported the average and standard deviation from these runs. To validate the performance of the predictors, an independent test dataset with 100 samples was additionally collected and labelled in the same way as the training set (Table S3). The ratios of the samples in each class was similar to the train set. The selected best predictor was then used to classify all the calculated pockets.

3. CaverDock Energy Analysis

3.1 Preparing CaverDock calculations

CaverDock 1.1 was used to analyse the ligand pathways in all cases in the dataset with successfully calculated tunnels. For each calculation, the receptor, ligand, and tunnel input files were prepared in several steps: (i) The ligand was prepared by automatic parsing of the annotated cognate reaction and cognate ligand as follows. The annotated KEGG [2] reaction was used to build the reaction scheme. Then all the reactants and product KEGG codes were parsed from the specific KEGG reaction website. Finally, using the molecule codes, the reaction scheme was written in a file (e.g., C01 C02 >> C03 C04). (ii) The molecule codes from the reaction scheme were used to parse the MOL file for each molecule. The MOL files were converted to SMILES codes, and the reaction was built based on the reaction scheme. (iii) The reaction in SMILES format was used as input in Reaction Decoder Tool 2.4.1 (RDT) [15]. The RDT analysed the reaction and annotated the atoms which changed in the molecules. The algorithm in RDT can annotate reactions where the reactants and products differ in atom composition. It fails on isomerisation reactions because there is no change in the number or type of atoms. Moreover, the algorithm is unable to work with polymeric structures because of the formatting in MOL files (such as cellulose). (iv) RDKit from *https://github.com/rdkit/rdkit* was used to decode the changes in the previously saved reaction and define the reaction centre for the molecules. (v) The information about the reaction centre was used for setting up the drag atom for CaverDock calculations. The drag atom is used to constrain the ligand to discs and pull the ligand through the tunnel. For reactants, the atom closest to the reaction centre was selected. In the case of products, the atom which was the most distant from the reaction centre was used. Pulling the ligand by the distant end of the molecule aims to better simulate the behaviour of leaving product molecule in the static snapshot of the protein structure. In the case of previous failure to annotate the reaction, the default settings were used (the atom closest to the molecule centroid). In lower-bound CaverDock trajectories the drag atom is constrained to the disc, but the rest of the molecule can move freely. The docking of the molecule is not

fully independent, therefore, the use of the explicitly set drag atom can improve the success rate of CaverDock calculations with large ligands with many degrees of freedom. (vi) The cognate ligand MOL file was converted to PDBQT by using the prepare-ligand4 script from MGLtools 1.5.6 [16]. (vii) Each tunnel was discretized using the Discretizer tool from the CaverDock package with default settings. (viii) The PDB of the biological unit was converted to PDBQT using the prepare-receptor4 script from MGLtools 1.5.6 [16]. (ix) Finally, the grid box around the tunnel and the configuration file were prepared by the prepare-config script from the CaverDock package. Only the lower-bound trajectory was calculated and analysed.

Several important energy values were extracted from the energy profiles manually for the validation dataset and automatically in the annotation pipeline. E_{Bound} is the minimum energy in the binding site, and it was extracted from the first half of the energy profile. E_{Max} is the maximum energy of the entire energy profile. For the collection of E_{Max} values, we did not consider the peak energy at the beginning of the energy profiles, which is likely to be an artefact caused by pushing the ligand too close to the bottom of the tunnel. These values were extracted manually in the case of the MD validation and later based on our testing of how often the artificial peak is present in the beginning of the profile, we excluded the first third (33 %) of the profiles automatically in the data analysis part. $E_{Surface}$ was taken from the last tunnel disk at the surface of the protein. Since the binding energy of the fully unbound ligand in AutoDock Vina is 0, in the case where $E_{Surface}$ was higher than 0, we changed it to 0. This is linked to the fact that if we could extend the tunnel enough, we would always approach 0 kcal/mol once the ligand had no interactions. If the surface energy was below 0, we kept this value due to the possibility of favourable binding at the surface of the protein. The energy barriers were then calculated as $E_a = E_{Max} - E_{Bound}$ for the products and $E_a = E_{Max} - E_{Surface}$ for the reactants.

3.2 Validation of CaverDock trajectories by molecular dynamics

The CaverDock tool has been tested extensively and used on various datasets in previous publications [17, 18]. However, validation of the quality of predicted trajectories from CaverDock has not been done by any method approaches based on Molecular Dynamics (MD). We explored several approaches for the simulation of ligand binding or unbinding, and in the end, we selected the Adaptive Steered Molecular Dynamics (ASMD) [19], because we were able to implement this method for our purpose. The ASMD method applies constant external force on two atoms in the simulated systems. This can be used to simulate unbinding or binding ligands through tunnels. The direction of the movement is set by selecting the steering atoms to move the ligand in the direction of a selected tunnel by lengthening or shortening the distance for unbinding or binding respectively. While changing the distance between those two atoms, the ligand moves in the given direction, but it can follow the curves of the tunnel which allows it to move through the protein. The steering atoms or the direction are not

restarted or changed during the simulation. To proceed with the validation, we selected eight cases that had 2-4 well-defined tunnels and the cognate product bound inside (Table S4). All the branches of the same tunnel apart from the highest--priority one were omitted. Seven out of the eight selected enzyme-cognate ligand systems are not part of the final filtered input dataset used for the automatic annotation and data analysis due to the updates of the input dataset based on changes in structural databases used to gather the data. These changes have had no impact on the comparison of CaverDock with MD simulations.

To prepare the complexes for the unbinding simulations, we selected the lowest-energy binding pose from the CaverDock analysis of the first tunnel. We also checked that the binding pose with similar energy and position could be found in the CaverDock trajectories from other tunnels. After extracting this starting pose, we added hydrogens to the molecule by Open Babel [20]. The RESP charges for ligands were calculated by Gaussian09_E.01 within R. E. D. Server [21]. The correct bond types were assigned by Antechamber, and Parmchk2 was used to generate the FCRMOD files [22].

The protein molecule was processed by Pdb4amber [22], and the protonation state of the protein was calculated by the H++ server at pH 7 and 0.1 M salinity [23]. The complex for MD simulation was then prepared with the following steps. The original crystallization solvent and the binding pose of the ligand were added, solvent molecules clashing with the position of the ligand were removed, and the *tLEaP* program of AmberTools 16 [22] was used to prepare the topology and coordinates files. The ff14SB force field [24] was applied in all simulations, Na^+ and Cl^- ions were added to neutralize the system and achieve a 0.1 M concentration of NaCl, and a truncated octahedral box of TIP3P [25] water molecules, with the edges at least 10 Å away from the protein atoms, was added.

The minimization and equilibration MDs were carried out with PMEMD.CUDA [26, 27] module of AMBER 16 [22]. In total, five minimization steps and twelve steps of equilibration dynamics were performed before the production of MDs. The first four minimization steps, composed of 2,500 cycles of steepest descent followed by 7 500 cycles of conjugate gradient, were performed as follows: (i) in the first one, all the atoms of the protein and ligand were restrained with a 500 kcal/mol·Å^2 harmonic force constant; (ii) in the following ones, only the backbone atoms of the protein and heavy atoms of the ligand were restrained, respectively, with 500, 125, and 25 kcal/mol·Å^2 force constants. A fifth minimization step, composed of 5,000 cycles of steepest descent and 15,000 cycles of conjugate gradient, was performed without any restraints. The subsequent MD simulations employed periodic boundary conditions, the particle mesh Ewald method for treatment of the long-range interactions beyond the 10 Å cut-off [28], the SHAKE algorithm [29] to constrain the bonds involving the hydrogen atoms, the Langevin thermostat with collision frequency 1.0 ps^{-1}, and a time step of 2 fs. Equilibration dynamics were performed in twelve steps: (i) 20 ps of gradual heating from 0 K to 300 K, under constant volume, restraining the protein atoms and ligand with a 200 kcal/mol·Å^2 harmonic force constant; (ii)

ten MDs of 400 ps each, at constant pressure (1 bar) and constant temperature (300 K), with gradually decreasing the restraints on the backbone atoms of the protein and atoms of the ligand with harmonic force constants of 150, 100, 75, 50, 25, 15, 10, 5, 1, and 0.5 kcal/mol·Å^2; (iii) 400 ps of unrestrained MD at the same conditions as the previous restrained MDs. The energy and coordinates were saved every 10 ps.

Before we started with the biased unbinding simulations, we ran classical MD simulations of *System #3* Cellobiohydrolase with Cellobiose and *System #4* Cytochrome P450 BM3 with 11,14,15-Trihydroxyicosatrienoic acid to showcase the need for steered MD and approximative methods for the study of ligand unbinding. We used the prepared complexes and ran 3 replicas of 1 μs simulations to study the behaviour of the complexes and potential unbinding of the ligand molecules.

Next, the unbinding trajectories were calculated with ASMD [19]. We used the settings obtained from the tutorial for AMBER. The following parameters were used: 25 parallel simulations, 2 Å stages, a velocity of 10 Å/ns, and a force of 7.2 N. The rest of the MD settings were set as in the last equilibration step. The protein atom for the steering was different for each tunnel. We selected the Cα atom in the residue at the bottom of the tunnel which was located opposite to the tunnel opening so that the ligand could be pushed from the binding site in this direction (Table S4). The ligand atom for steering was selected as the one closest to the centroid of the molecule. This setting was used in all systems, except for the *System #8* with UDP-glucosyltransferase and Uridine 5'-diphosphate: Due to the size and shape of this ligand, we were unable to simulate any trajectories using the centroid atom. In this case, we had to select the atom closest to the tunnel for each unbinding simulation. The selenium atom in Se-Adenosyl-L-selenohomocysteine of *System #5* was changed to sulphur in ASMD simulations. To compare the ASMD simulations with CaverDock, we extended the CaverDock trajectories to match the length of the simulated distances from ASMD.

Apart from the ASMD simulations, we ran MD simulations with ligand-free structures to generate ensembles of protein snapshots to study how much CaverDock results change when using dynamical structures. We used the same settings for the preparation of the systems, minimisation, and equilibration. We ran 50 ns of production MD, saved every 1,000th snapshot, from these 25,000 snapshosts we collected 100 snapshots covering the entire MD simulation. The solvent molecules were deleted, and the snapshots were aligned and centred to the first snapshot with Cpptraj [22]. We calculated the tunnels in selected snapshots using CAVER 3.02 [11] with a smaller probe radius of 0.5 Å. The rest of the setup was the same as during the annotation. Tunnel clusters found in MD snapshots were visually matched with the tunnels found in the static structures. Using the same workflow for CaverDock simulations as in the annotation, we calculated the transport of ligands through the snapshots. The tunnels were discretized and extended by 5 Å. CaverDock calculations were run with the drag atoms defined in the annotation pipeline. Finally, we averaged the energy values for each tunnel in every

system. The Potential of Mean Force profiles from ASMD and CaverDock energy profiles from a single static structure and averaged values were then compared. We are aware that both MDs and CaverDock use different methods for parametrisation. Our main aim was the qualitative comparison to see if the molecules can unbind through the selected tunnels, and whether the order of energy profiles matches.

References

1. Tyzack JD, Fernando L, Ribeiro AJM, Borkakoti N, Thornton JM. Ranking Enzyme Structures in the PDB by Bound Ligand Similarity to Biological Substrates. Structure. 2018;26:565-571.e3.

2. Kanehisa M, Goto S. KEGG: kyoto encyclopedia of genes and genomes. Nucleic Acids Res. 2000;28:27–30.

3. Sumbalova L, Stourac J, Martinek T, Bednar D, Damborsky J. HotSpot Wizard 3.0: web server for automated design of mutations and smart libraries based on sequence input information. Nucleic Acids Res. 2018;46:W356–62.

4. Consortium U. UniProt: the universal protein knowledgebase. Nucleic Acids Res. 2017;45:D158–69.

5. Furnham N, Holliday GL, de Beer TAP, Jacobsen JOB, Pearson WR, Thornton JM. The Catalytic Site Atlas 2.0: cataloging catalytic sites and residues identified in enzymes. Nucleic Acids Res. 2014;42:D485–9.

6. Shrake A, Rupley JA. Environment and exposure to solvent of protein atoms. Lysozyme and insulin. J Mol Biol. 1973;79.

7. Prlić A, Yates A, Bliven SE, Rose PW, Jacobsen J, Troshin P V., et al. BioJava: an open-source framework for bioinformatics in 2012. Bioinformatics. 2012;28:2693–5.

8. Gutmanas A, Alhroub Y, Battle GM, Berrisford JM, Bochet E, Conroy MJ, et al. PDBe: Protein Data Bank in Europe. Nucleic Acids Res. 2014;42 Database issue:D285-91.

9. Le Guilloux V, Schmidtke P, Tuffery P. Fpocket: an open source platform for ligand pocket detection. BMC Bioinformatics. 2009;10:168.

10. Fischer JD, Holliday GL, Thornton JM. The CoFactor database: organic cofactors in enzyme catalysis. Bioinformatics. 2010;26:2496–7.

11. Chovancova E, Pavelka A, Benes P, Strnad O, Brezovsky J, Kozlikova B, et al. CAVER 3.0: a tool for the analysis of transport pathways in dynamic protein structures. PLoS Comput Biol. 2012;8:e1002708.

12. Rose PW, Beran B, Bi C, Bluhm WF, Dimitropoulos D, Goodsell DS, et al. The RCSB Protein Data Bank: redesigned web site and web services. Nucleic Acids Res. 2011;39 Database issue:D392-401.

13. Ma J, Wang S. Algorithms, applications, and challenges of protein structure alignment. Adv Protein Chem Struct Biol. 2014;94:121–75.

14. Pratt JW, Gibbons JD. Kolmogorov-Smirnov Two-Sample Tests. 1981. p. 318–44.

15. Rahman SA, Torrance G, Baldacci L, Martínez Cuesta S, Fenninger F, Gopal N, et al. Reaction Decoder Tool (RDT): extracting features from chemical reactions. Bioinformatics. 2016;32:2065–6.

16. Morris GM, Huey R, Lindstrom W, Sanner MF, Belew RK, Goodsell DS, et al. AutoDock4 and AutoDockTools4: Automated docking with selective receptor flexibility. J Comput Chem. 2009;30:2785–91.

17. Pinto GP, Vavra O, Filipovic J, Stourac J, Bednar D, Damborsky J. Fast Screening of Inhibitor Binding/Unbinding Using Novel Software Tool CaverDock. Front Chem. 2019;7.

18. Pinto GP, Vavra O, Marques SM, Filipovic J, Bednar D, Damborsky J. Screening of world approved drugs against highly dynamical spike glycoprotein of SARS-CoV-2 using CaverDock and machine learning. Comput Struct Biotechnol J. 2021;19:3187–97.

19. Ozer G, Quirk S, Hernandez R. Adaptive steered molecular dynamics: validation of the selection criterion and benchmarking energetics in vacuum. J Chem Phys. 2012;136:215104.

20. O'Boyle NM, Banck M, James CA, Morley C, Vandermeersch T, Hutchison GR. Open Babel: An open chemical toolbox. J Cheminform. 2011;3:33.

21. Vanquelef E, Simon S, Marquant G, Garcia E, Klimerak G, Delepine JC, et al. R.E.D. Server: a web service for deriving RESP and ESP charges and building force field libraries for new molecules and molecular fragments. Nucleic Acids Res. 2011;39 suppl:W511–7.

22. Case DA, Cheatham TE, Darden T, Gohlke H, Luo R, Merz KM, et al. The Amber biomolecular simulation programs. J Comput Chem. 2005;26:1668–88.

23. Gordon JC, Myers JB, Folta T, Shoja V, Heath LS, Onufriev A. H++: a server for estimating pKas and adding missing hydrogens to macromolecules. Nucleic Acids Res. 2005;33 Web Server issue:W368-71.

24. Maier JA, Martinez C, Kasavajhala K, Wickstrom L, Hauser KE, Simmerling C. ff14SB: Improving the Accuracy of Protein Side Chain and Backbone Parameters from ff99SB. J Chem Theory Comput. 2015;11:3696–713.

25. Jorgensen WL, Chandrasekhar J, Madura JD, Impey RW, Klein ML. Comparison of simple potential functions for simulating liquid water. J Chem Phys. 1983;79:926–35.

26. Salomon-Ferrer R, Götz AW, Poole D, Le Grand S, Walker RC. Routine Microsecond Molecular Dynamics Simulations with AMBER on GPUs. 2. Explicit Solvent Particle Mesh Ewald. J Chem Theory Comput. 2013;9:3878–88.

27. Le Grand S, Götz AW, Walker RC. SPFP: Speed without compromise—A mixed precision model for GPU accelerated molecular dynamics simulations. Comput Phys Commun. 2013;184:374–80.

28. Darden T, York D, Pedersen L. Particle mesh Ewald: An N ·log(N) method for Ewald sums in large systems. J Chem Phys. 1993;98:10089–92.

29. Ryckaert J-P, Ciccotti G, Berendsen HJ. Numerical integration of the cartesian equations of motion of a system with constraints: molecular dynamics of n-alkanes. J Comput Phys. 1977;23:327–41.

Supplementary tables

Table S1: List of features used for training pocket annotation predictor

Feature Origin	Feature
Fpocket	Fpocket Score
	Druggability Score
	Number of Alpha Spheres
	Total Surface Area
	Polar Surface Area
	Apolar Surface Area
	Volume
	Mean local hydrophobic density
	Mean alpha sphere radius
	Mean alpha sphere solvent access
	Proportion of apolar alpha spheres
	Hydrophobicity score
	Volume score
	Polarity score
	Charge score
	Proportion of polar atoms
	Density of the cavity
	Maximum distance between two alpha spheres
	Bfactor score
Custom	Exposed ratio

Table S2: List of hyperparameters of algorithms and their values

Algorithms	Hyperparameters	Values		Tested ranges
		Two-class dataset	Three-class dataset	
KNN	K (Number of neighbors)	17	6	1..30
Random Forest	max_depth (maximum depth of trees)	2	2	1..5
	n_estimators (number of trees)	4	4	1..5
SVM	Kernel	Linear	Linear	['rbf', 'poly', 'linear']
	C (regularization parameter)	0.13	0.14	-3..0 (Logarithm with 50 steps)
Shallow ANN	Alpha (L2 penalty)	0.1	1	-1..0 (Logarithm with 10 steps)
	Hidden_layer_sizes (number of hidden layer neurons)	43	21	2..64
	Activation function	tanh	tanh	['identity', 'logistic', 'tanh', 'relu']
	Solver	sgd	sgd	['lbfgs', 'sgd', 'adam']

Table S3: The structure of the training and test data based on the labels.

	# Data points	# Surface (1)	# Borderline (0)	# Buried (-1)
**Train set**	200	92	58	50
**Test set**	100	40	30	30

Table S4: Validation systems and applied settings for simulations.

PDB-ID	Tunnel	ASMD settings		CaverDock settings
		Protein residue	Ligand atom	Drag atom
System #1 (PDB ID 1OTW)	1	A MET 221	C9	26
	2	A LYS 214		
	3	A THR 73		
System #2 (PDB ID 2BFN)	1	GLY 18	C2	1
	2	ARG 191		
	3	LEU 177		
System #3 (PDB ID 2RFY)	1	ARG 129	O1	default
	2	PRO 249		
	3	THR 248		
System #4 (PDB ID 2UWH)	1	THR 269	C12	10
	2	ASP 69		
	3	LEU 182		
System #5 (PDB ID 4E2Z)	1	HIS 181	N	default
	2	ILE 390		
	3	GLY 138		
System #6 {PDB ID 5EDT)	1	THR 243	C7	default
	2	ALA 349		
	3	HEME		
	4	LEU 209		
System #7 (PDB ID 3ORW)	1	LEU 130	C1	12
	2	ALA 251		
System #8 (PDB ID 5U6M)	1	ASN 346	P1	default
	2	PHE 354	C	
	3	ARG 28	C6	

Table S5: Percentages of pockets with the best Fpocket and druggability scores.

Subset	Pocket type based on ligand coverage	Number of cases	Ligand-binding pocket has best Fpocket score [%]	Ligand-binding pocket has best Druggability score [%]
**Pockets with matching annotations**	All pockets	8,350	42.83	22.50
	Single pocket	3,344	49.85	25.48
	One main pocket	4,262	39.32	21.52
	Two main pockets	744	31.32	14.78
**Pockets with no matching residue annotations**	All pockets	2,696	26.97	12.20
	Single pocket	941	30.50	14.45
	One main pocket	1,464	26.98	10.93
	Two main pockets	291	15.46	11.34
**Pockets with no annotations**	All pockets	4,651	40.94	17.37
	Single pocket	1,682	50.65	20.15
	One main pocket	2,528	37.46	16.30
	Two main pockets	441	23.81	12.93

Table S6: The performance of five selected predictors on training and test data. The reported values on the training set are based on the 5-fold cross-validation. All the values are given as averages and standard deviations based on 100 independent runs.

Dataset	Method	Accuracy		F1		1-FPR	
		Training set	Test set*	Training set	Test set*	Training set	Test set*
Three-class problem	KNN (k=6)	0.51 ± 0.05	0.47	0.47 ± 0.05	0.45	0.70 ± 0.12	**0.75**
	SVM	**0.56 ± 0.05**	0.52	0.49 ± 0.05	0.44	0.69 ± 0.11	0.65
	Random Forest	0.48 ± 0.03	0.48 ± 0.04	0.51 ± 0.04	0.40 ± 0.05	0.43 ± 0.13	0.37 ± 0.16
	ANN (21 neurons)	0.55 ± 0.02	**0.54 ± 0.02**	**0.63 ± 0.02**	**0.50 ± 0.03**	0.70 ± 0.04	0.67 ± 0.05
	Naïve Bayesian	0.34 ± 0.05	0.40	0.30 ± 0.05	0.19	**0.90 ± 0.10**	0.0
Two-class problem	KNN (k=17)	0.67 ± 0.04	**0.71**	0.65 ± 0.04	**0.71**	0.79± 0.05	0.70
	SVM	**0.70 ± 0.04**	0.70	0.69 ± 0.04	0.70	0.70 ± 0.06	0.62
	Random Forest	0.61 ± 0.03	0.66 ± 0.04	0.72 ± 0.02	0.65 ± 0.04	0.78 ± 0.06	0.68 ± 0.07
	ANN (43 neurons)	0.66 ± 0.02	0.70 ± 0.02	**0.74 ± 0.02**	0.70 ± 0.02	0.76 ± 0.02	**0.70 ± 0.03**
	Naïve Bayesian	0.57 ± 0.04	0.41	0.45 ± 0.05	0.41	**0.93 ± 0.05**	0.02

*KNN, SVM and Naïve Bayesian did not have any randomization, which we could use to have the standard deviation on the test set.

Table S7: CaverDock energies in static structures and snapshots from molecular dynamics.

CaverDock energies in static structure				Priority in ASMD	Averaged CD energies from 100 snapshots			
Case	Tunnel	Priority in PDB	E_{max} [kcal/mol]		Priority in MD	E_{max} [kcal/mol]	Finished calculations	Tunnel occurrence*
System 1 (1OTW)	1	1	20.4	2	3	-1.3	13	13
	2	2	21.8	1	1	1.3	25	25
	3	3	46.6	3	5	15.4	8	8
System 2 (2BFN)	1	1	-2.5	1	1	-1.9	95	100
	2	3	1.7	2	2	-1.2	78	78
	3	4	6.2	3	None	None	0	0
System 3 (2RFY)	1	1	-3.3	1	1	-3.5	61	69
	2	2	-5.5	3	3	-0.9	56	60
	3	3	-1.9	2	2	-4.6	58	62
System 4 (2UWH)	1	1	-4.9	1	3	-3.5	81	85
	2	2	2.8	2	1	-4.1	78	79
	3	4	3.9	3	6	-0.2	16	16
System 5 (4E2Z)	1	1	-1.4	1	1	-4.7	73	74
	2	2	7.8	2	2	1.2	84	89
	3	5	48.0	3	30	15.4	4	4
System 6 {5EDT)	1	1	28.7	2	1	10.5	60	60
	2	2	47.0	1	4	24.3	26	26
	3	3	19.2	3	3	15.1	47	47
	4	5	39.6	4	8	22.5	15	15
System 7 (3ORW)	1	1	-0.6	1	1	-3.8	96	96
	2	3	12.0	2	5	10.5	9	9
System 8 (5U6M)	1	1	-6.2	1	1	-5.4	90	92
	2	2	-2.8	2	4	-4.2	87	89
	3	4	31.1	3	5	12.4	33	33

* number of snapshots out of 100 containing the tunnel

Table S8: Length of the first three tunnels in predicted classes.

Tunnel number	Predicted pocket class	Cases	Tunnel length		
			x < 5 Å [%]	5 Å < x < 15 Å [%]	15 Å < x [%]
Tunnel 1	Buried	3,552	25.11	45.97	28.91
	Borderline	3,178	41.19	49.06	9.75
	Surface	7,702	74.24	22.44	3.32
Tunnel 2	Buried	3,215	2.33	40.34	57.33
	Borderline	2,798	2.64	61.62	35.74
	Surface	6,142	6.61	70.55	22.84
Tunnel 3	Buried	2,860	0.59	20.80	78.60
	Borderline	2,268	0.35	35.89	63.76
	Surface	4,907	1.35	44.37	54.29

Table S9: Match between energetic maximum and geometrical bottleneck.

Tunnels	Cases	E_{Max} in tunnel bottleneck			
		Bottleneck disc [%]	Bottleneck area 1.5 Å [%]	Bottleneck area 3 Å [%]	Bottleneck area 5 Å [%]
Tunnels 1-3	29 693	50.35	63.91	70.39	74.49
Tunnel 1	12 774	69.65	77.15	80.37	82.25
Tunnel 2	9 449	39.68	55.54	63.51	68.41
Tunnel 3	7 470	30.86	51.85	62.05	68.90

*data was collected from energy profiles with 33 % deleted parts

Table S10: Comparison of first three tunnels based on the energetic maximum.

Type of data	Number of cases
Protein-ligand pairs for CaverDock calculations	14,432
Successful CaverDock calculations	13,158
Failed CaverDock calculations	1,274
Tunnel 1 is the best tunnel in comparison [%]	**74.75**
Tunnel 2 is the best tunnel in comparison [%]	18.52
Tunnel 3 is the best tunnel in comparison [%]	6.73

Table S11: RMSD values for 50 ns MD simulations without ligand and ASMD simulations with ligands

System	Simulation	Average RMSD[a]	Standard deviation
System #1 (PDB ID 1OTW)	MD without ligand	1.53	0.14
	ASMD Tunnel 1	1.17	0.08
	ASMD Tunnel 2	1.43	0.15
	ASMD Tunnel 3	3.14	1.68
System #2 (PDB ID 2BFN)	MD without ligand	0.88	0.09
	ASMD Tunnel 1	0.82	0.09
	ASMD Tunnel 2	0.87	0.08
	ASMD Tunnel 3	0.80	0.07
System #3 (PDB ID 2RFY)	MD without ligand	1.24	0.08
	ASMD Tunnel 1	1.24	0.09
	ASMD Tunnel 2	1.36	0.09
	ASMD Tunnel 3	1.22	0.13
System #4 (PDB ID 2UWH)	MD without ligand	1.46	0.20
	ASMD Tunnel 1	1.33	0.15
	ASMD Tunnel 2	1.33	0.13
	ASMD Tunnel 3	3.93	2.44
System #5 (PDB ID 4E2Z)	MD without ligand	1.37	0.33
	ASMD Tunnel 1	1.25	0.29
	ASMD Tunnel 2	4.53	2.54
	ASMD Tunnel 3	3.66	2.09
System #6 (PDB ID 5EDT)	MD without ligand	1.43	0.13
	ASMD Tunnel 1	1.25	0.16
	ASMD Tunnel 2	1.35	0.16
	ASMD Tunnel 3	1.20	0.12
	ASMD Tunnel 4	3.80	2.09
System #7 (PDB ID 3ORW)	MD without ligand	1.20	0.10
	ASMD Tunnel 1	1.14	0.12
	ASMD Tunnel 2	1.94	0.49
System #8 (PDB ID 5U6M)	MD without ligand	1.64	0.20
	ASMD Tunnel 1	1.42	0.10
	ASMD Tunnel 2	1.73	0.26
	ASMD Tunnel 3	2.09	0.74

[a] The high RMSDs in some ASMD simulations correspond well with the observations presented in the results section 3.3.2. In these cases, high forces were applied to the drag atoms while the ligand got stuck or unbound through an incorrect tunnel. This impacted the conformation of the protein structure since the ligand was unable to pass through the narrow tunnels.

Supplementary figures

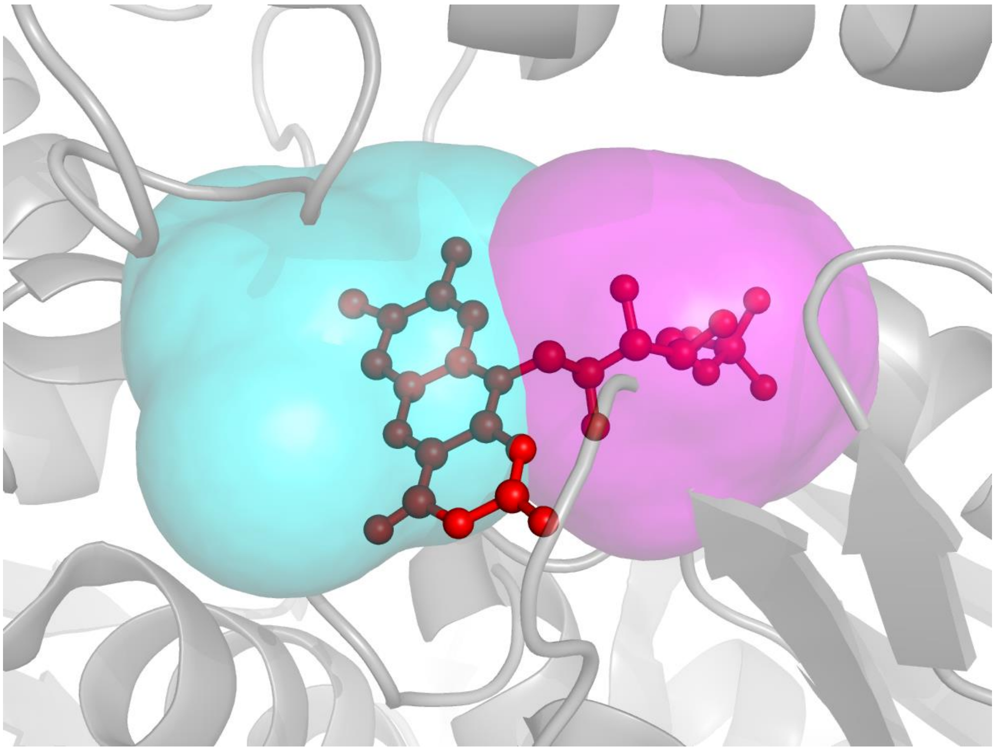

Figure S1: Visualisation of a flavin mononucleotide molecule, which is part of two main pockets in the structure of oxidoreductase FprA (PDB ID 1YCH). The first pocket (cyan) contains 52 % of the atoms of the bound ligand (red balls and sticks), and the second pocket (magenta) contains 48 % of the atoms. We illustrate how the settings for Fpocket 2 caused the algorithm to define the empty space as two pockets. Due to the low difference in ligand coverage, we selected pocket 1 as the main one based on the druggability score. This pocket does not contain any annotated residues, therefore, we did not have any correct matches.

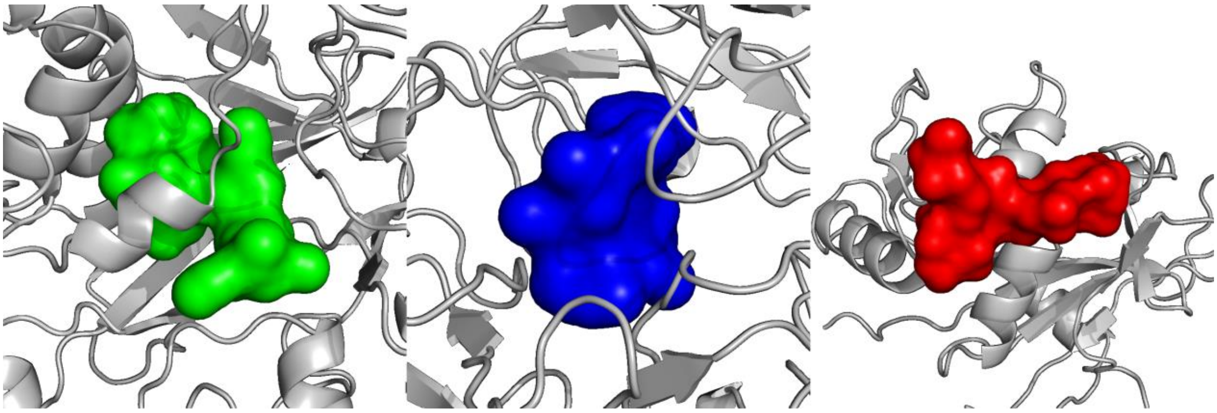

Figure S2: Illustration of the three classes of pockets based on the manual labelling. Example of a buried pocket (green) in structure 4RPJ, a borderline pocket (blue) in structure 2RK2 and a surface pocket (red) in structure 3NZB. The buried pocket is hidden inside of the protein and the bottom of the pocket is not easily accessible. In the case of the borderline pocket, it is not present at the surface of the protein but in a large channel-like opening inside of the protein and is easily reachable by ligands. Therefore, we classified it as borderline, something in between the other two classes of pockets. In the third example the pocket is located directly at the surface of the protein.

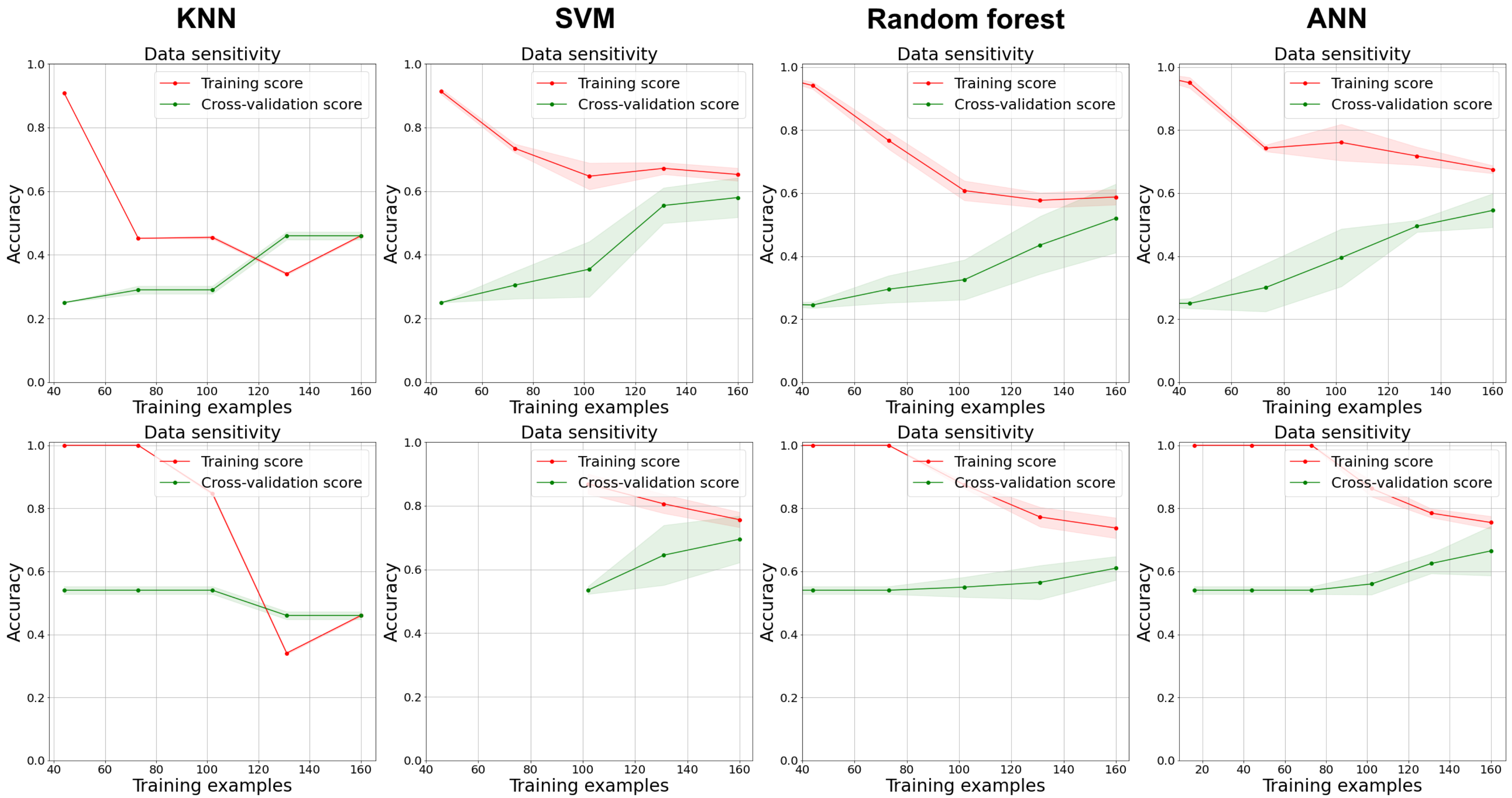

Figure S3: Learning curves for the predictors on the three-class (top) and two-class (bottom) problems. The labels on the x-axis show the number of training data points used at each fold, up to 160 data points in the case of the entire training dataset. For KNN, ANN, and SVMs, the convergence of the two curves indicates a good fit, suggesting that adding more data is unlikely to improve accuracy. In contrast, the gap between the training and cross-validation curves for the Random Forest shows slight overfitting but trends towards similar accuracy values of 50% and 70% for the three-class and two-class problems, respectively.

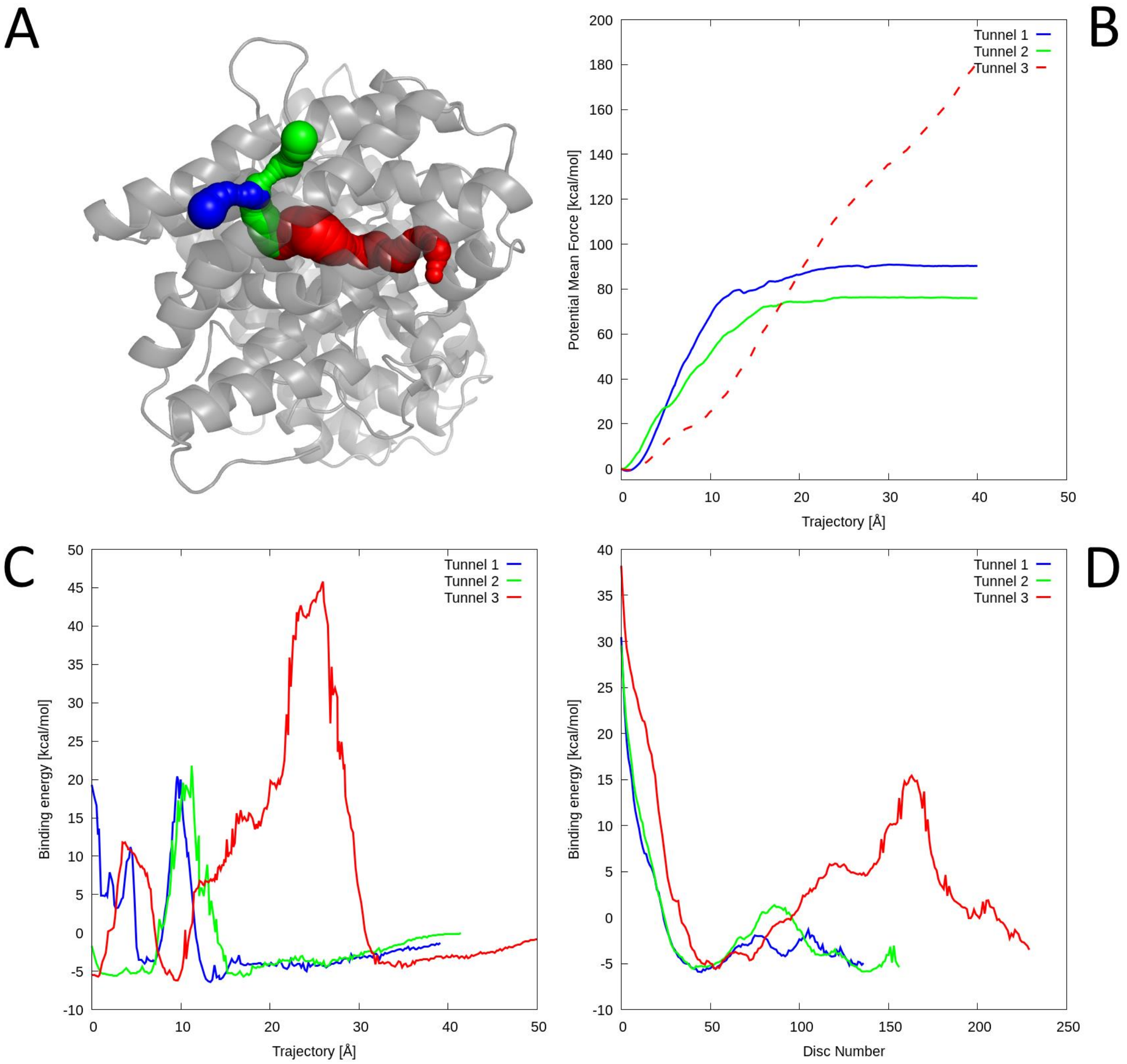

Figure S4: Results from CaverDock validation for System #1 - pyrroloquinoline-quinone synthase with pyrrolo-quinoline quinone. (A) Visualisation of the protein structure (PDB ID 1OTW) with analysed tunnels showed as spheres: tunnel 1 (blue), tunnel 2 (green), tunnel 3 (red); (B) Potential of Mean Force (PMF) profiles from ASMD simulations, the profile for stuck ligand is shown as dashed line; (C) energy profiles from static CaverDock calculations; (D) averaged CaverDock energy profiles from 50 ns simulation snapshots.

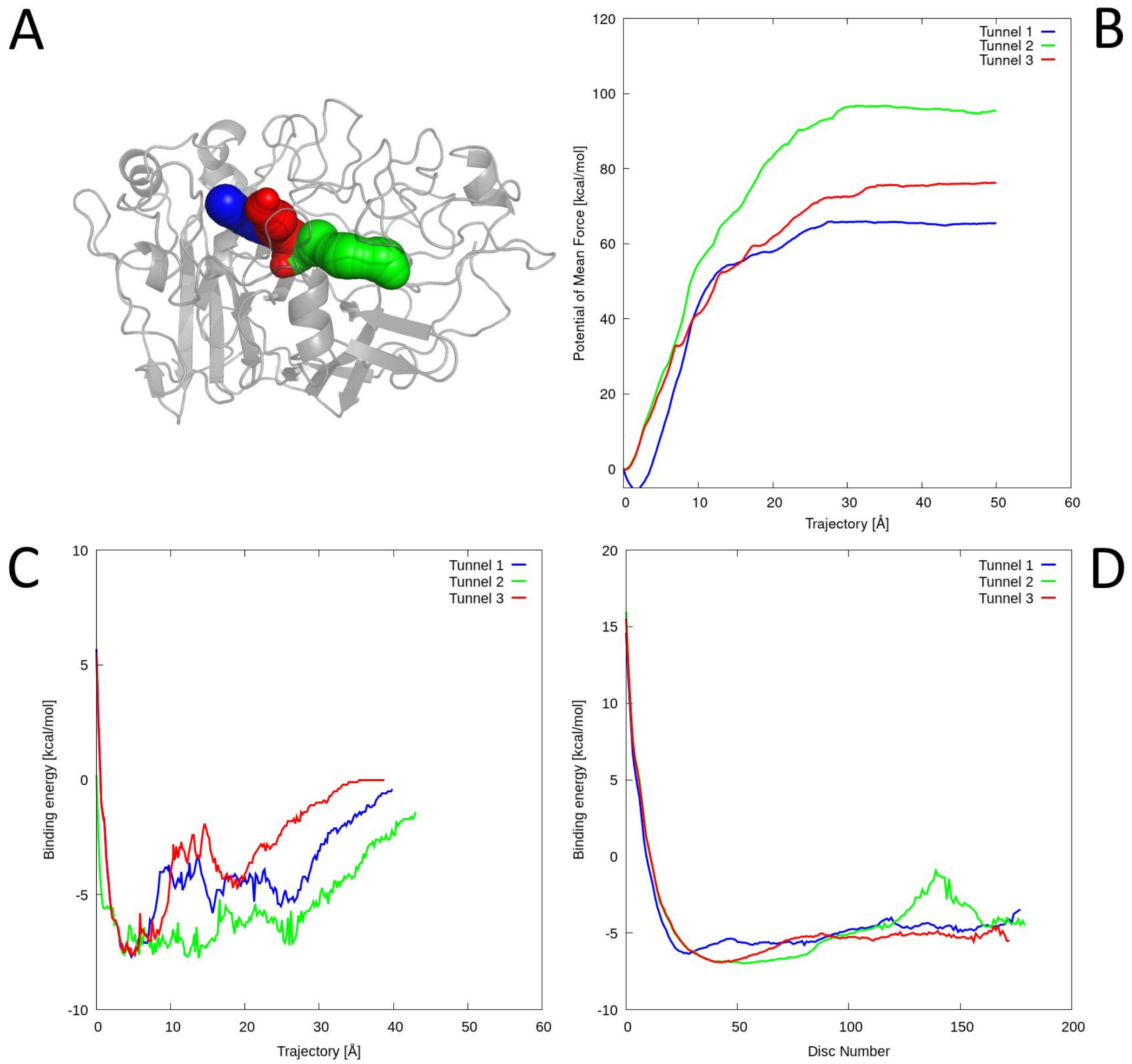

Figure S5: Results from CaverDock validation for System #3 - cellobiohydrolase with cellobiose. (A) Visualisation of the protein structure (PDB ID 2RFY) with analysed tunnels showed as spheres: tunnel 1 (blue), tunnel 2 (green), tunnel 3 (red); (B) PMF profiles from ASMD simulations; (C) energy profiles from static CaverDock calculations; (D) averaged CaverDock energy profiles from 50 ns simulation snapshots.

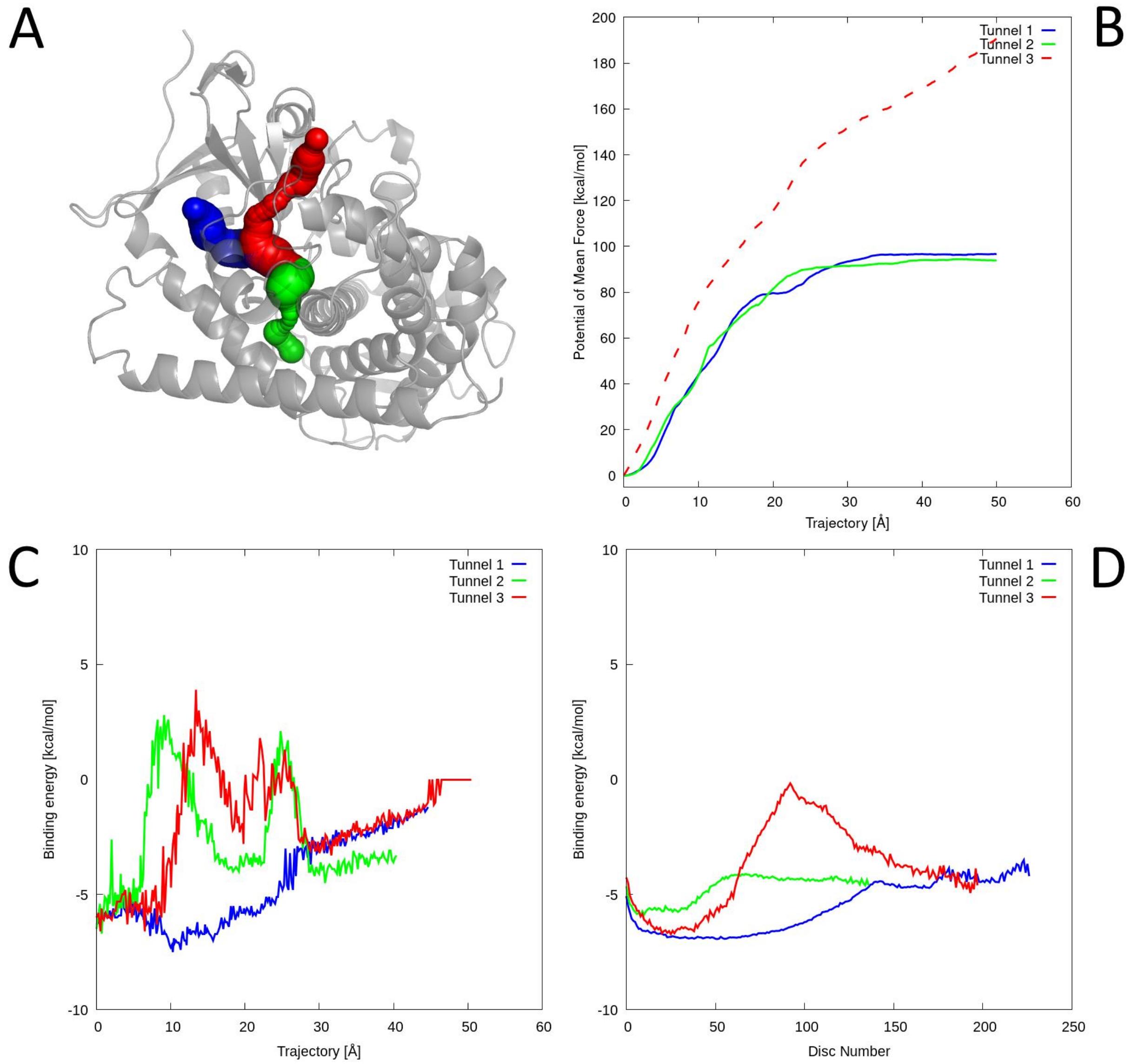

Figure S6: Results from CaverDock validation for System #4 - Cytochrome P450 BM3 with 11,14,15-trihydroxyicosatrienoic acid. (A) Visualisation of the protein structure (PDB ID 2UWH) with analysed tunnels showed as spheres: tunnel 1 (blue), tunnel 2 (green), tunnel 3 (red); (B) PMF profiles from ASMD simulations, the profile for stuck ligand is shown as dashed line; (C) energy profiles from static CaverDock calculations; (D) averaged CaverDock energy profiles from 50 ns simulation snapshots.

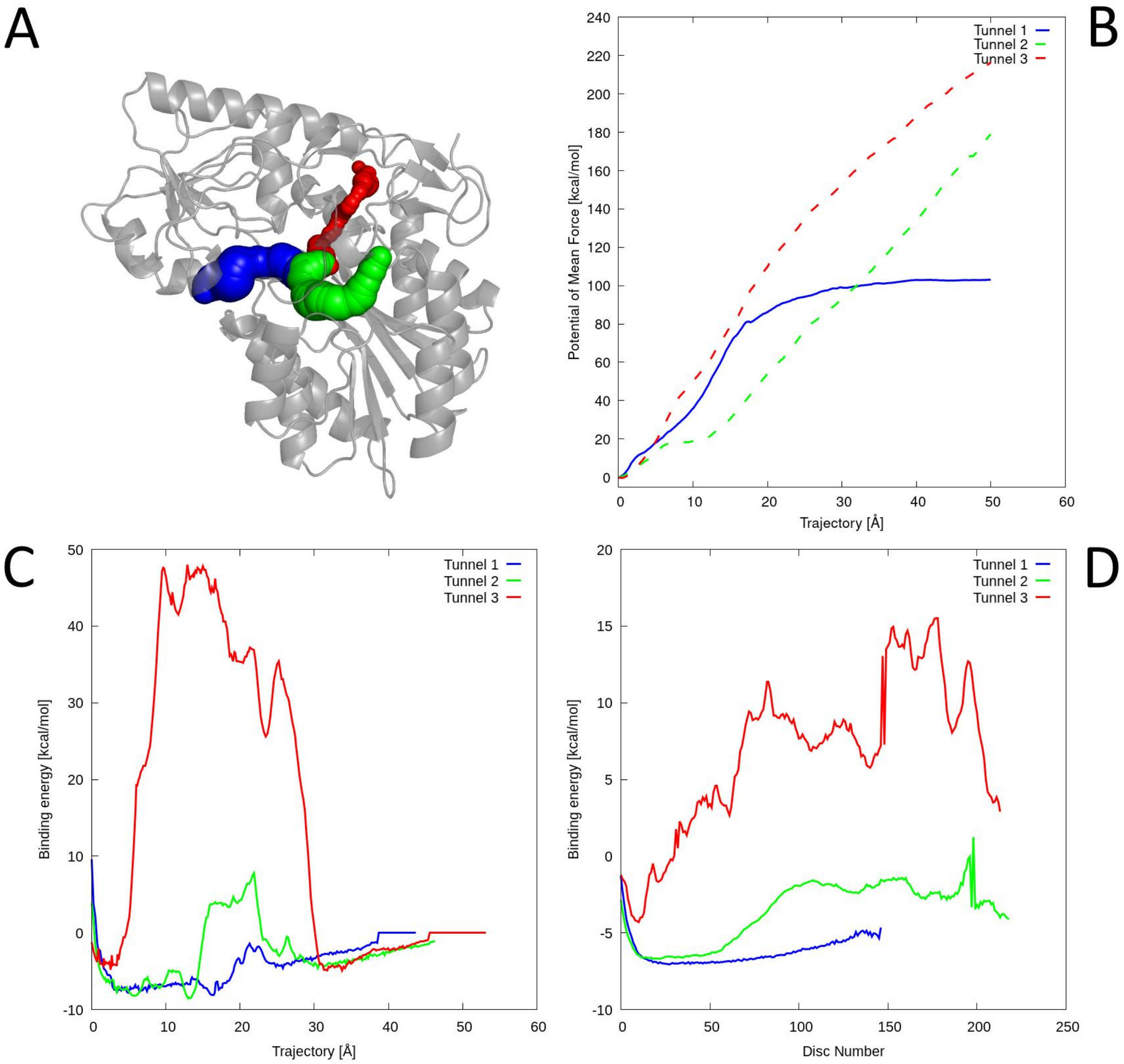

Figure S7: Results from CaverDock validation for System #5 - C-3'-methyltransferase with Se-adenosyl-L-selenohomocysteine. (A) Visualisation of the protein structure (PDB ID 4E2Z) with analysed tunnels showed as spheres: tunnel 1 (blue), tunnel 2 (green), tunnel 3 (red); (B) PMF profiles from ASMD simulations, the profiles for stuck ligand are shown as dashed line; (C) energy profiles from static CaverDock calculations; (D) averaged CaverDock energy profiles from 50 ns simulation snapshots.

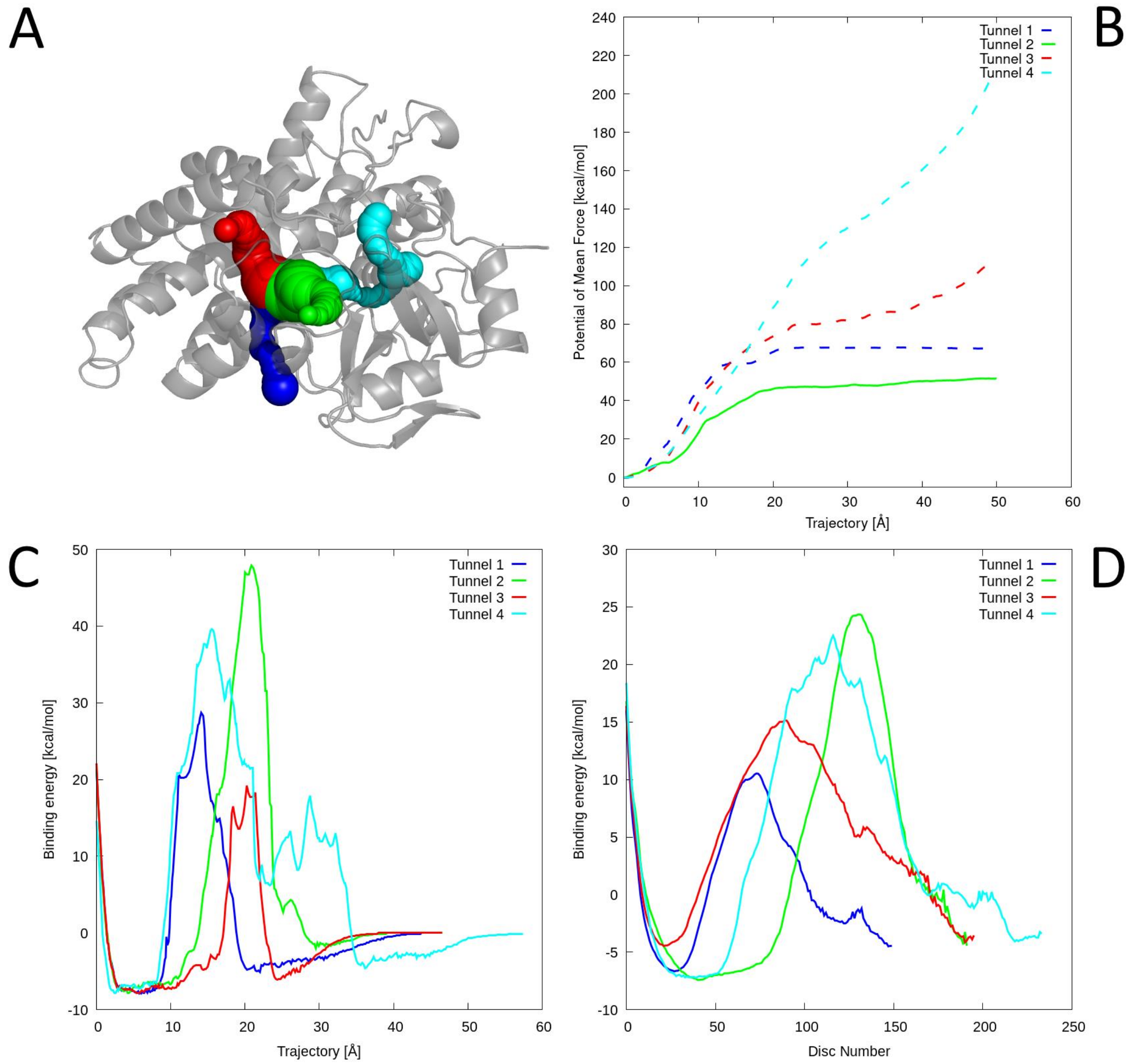

Figure S8: Results from CaverDock validation for System #6 - Cytochrome P450 CYP121 with (4*S*)-4-(5,5-Dimethylcyclohex-1-en-1-yl)cyclohex-1-ene-1-carboxylate. (A) Visualisation of the protein structure (PDB ID 5EDT) with analysed tunnels showed as spheres: tunnel 1 (blue), tunnel 2 (green), tunnel 3 (red), tunnel 4 (cyan); (B) PMF profiles from ASMD simulations, the profiles where ligand unbound through incorrect tunnel are shown as dashed line; (C) energy profiles from static CaverDock calculations; (D) averaged CaverDock energy profiles from 50 ns simulation snapshots.

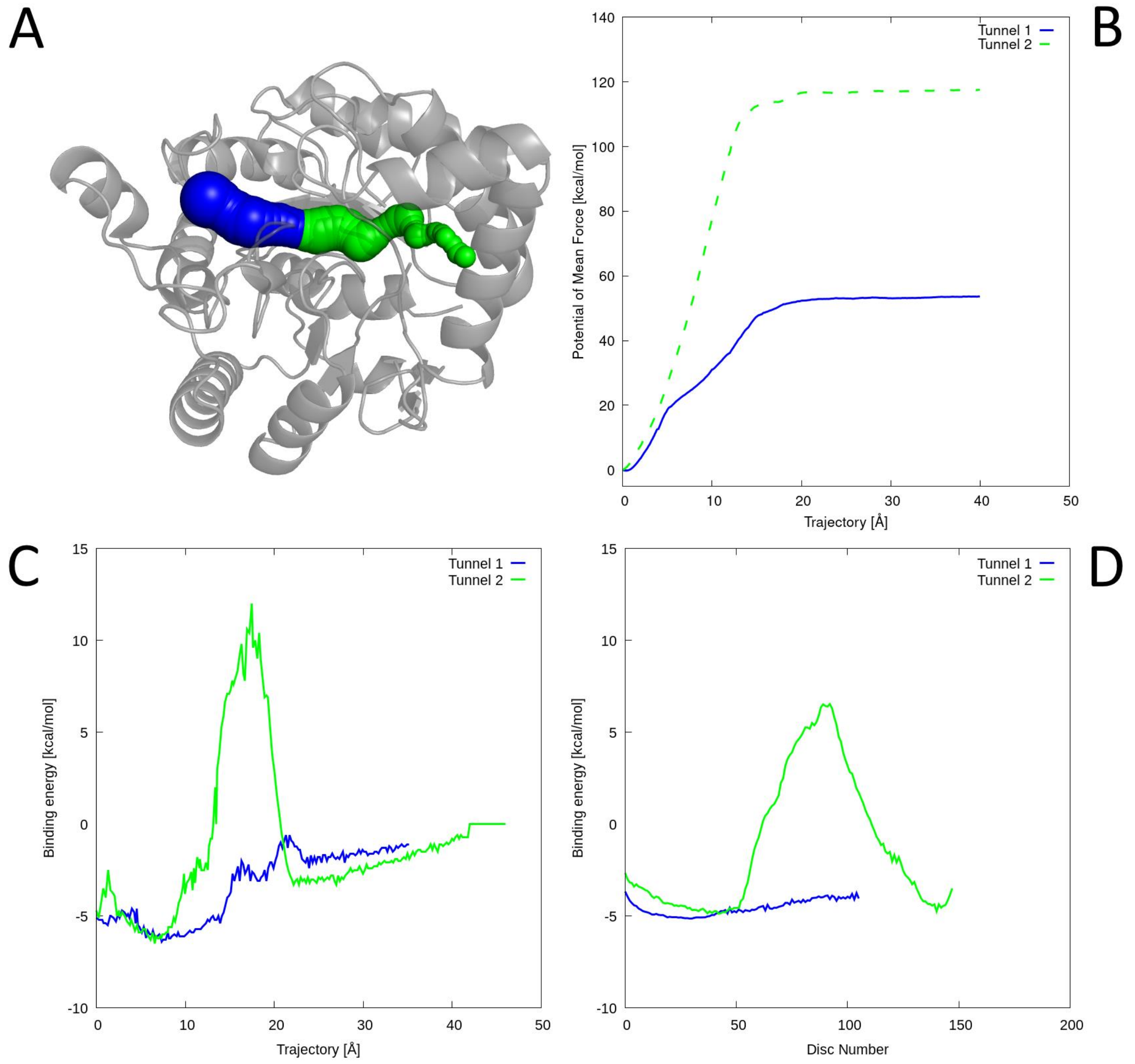

Figure S9: Results from CaverDock validation for System #7 – phosphotriesterase with N-(6-aminohexanoyl)-6-aminohexanoate. (A) Visualisation of the protein structure (PDB ID 3ORW) with analysed tunnels showed as spheres: tunnel 1 (blue), tunnel 2 (green); (B) PMF profiles from ASMD simulations, the profile where ligand unbound through incorrect tunnel is shown as dashed line; (C) energy profiles from static CaverDock calculations; (D) averaged CaverDock energy profiles from 50 ns simulation snapshots.

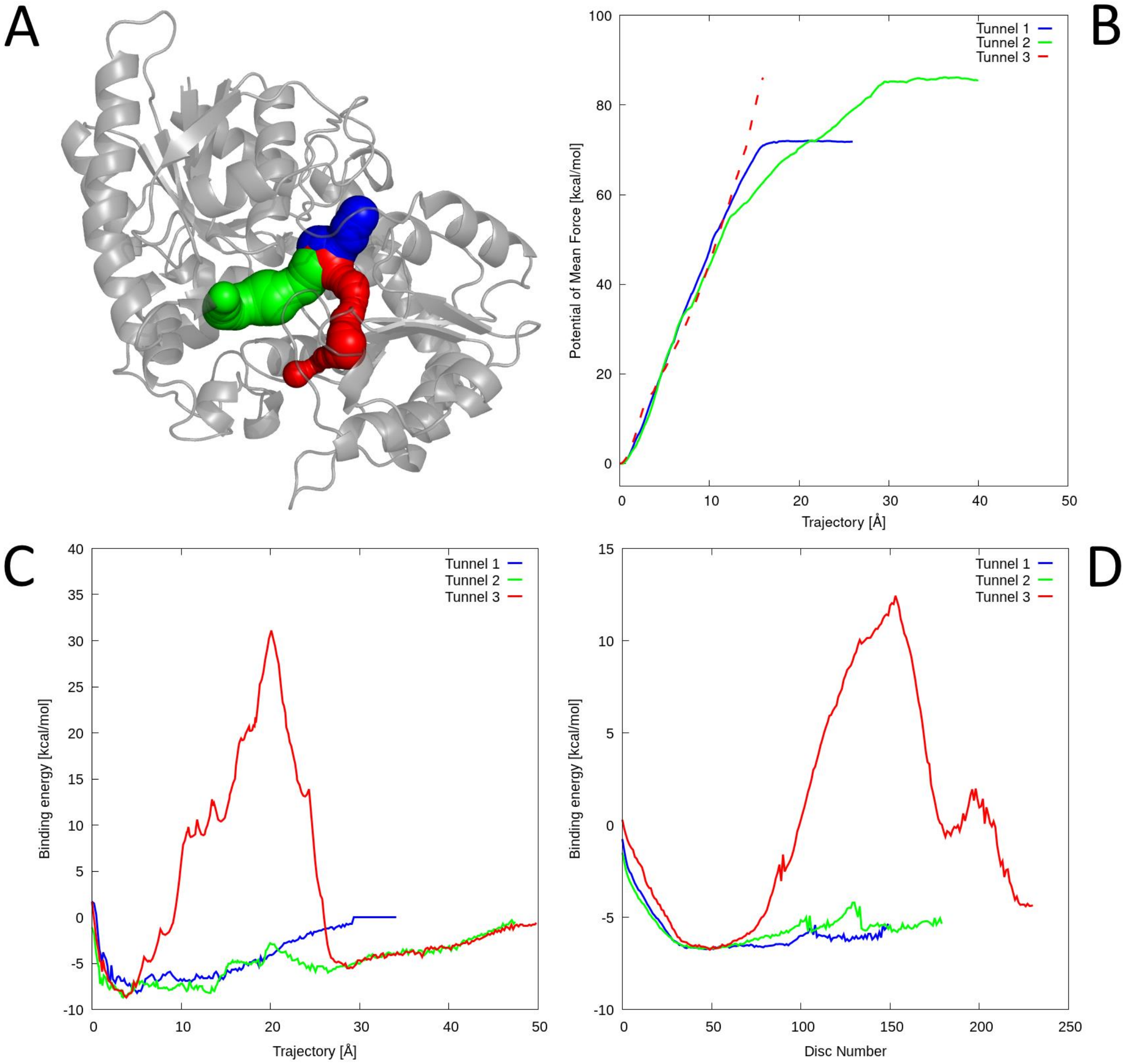

Figure S10: Results from CaverDock validation for System #8 – UDP-glucosyltransferase with uridine 5'-diphosphate. (A) Visualisation of the protein structure (PDB ID 5U6M) with analysed tunnels showed as spheres: tunnel 1 (blue), tunnel 2 (green), tunnel 3 (red); (B) PMF profiles from ASMD simulations, the profile for stuck ligand is shown as dashed line; (C) energy profiles from static CaverDock calculations; (D) averaged CaverDock energy profiles from 50 ns simulation snapshots.

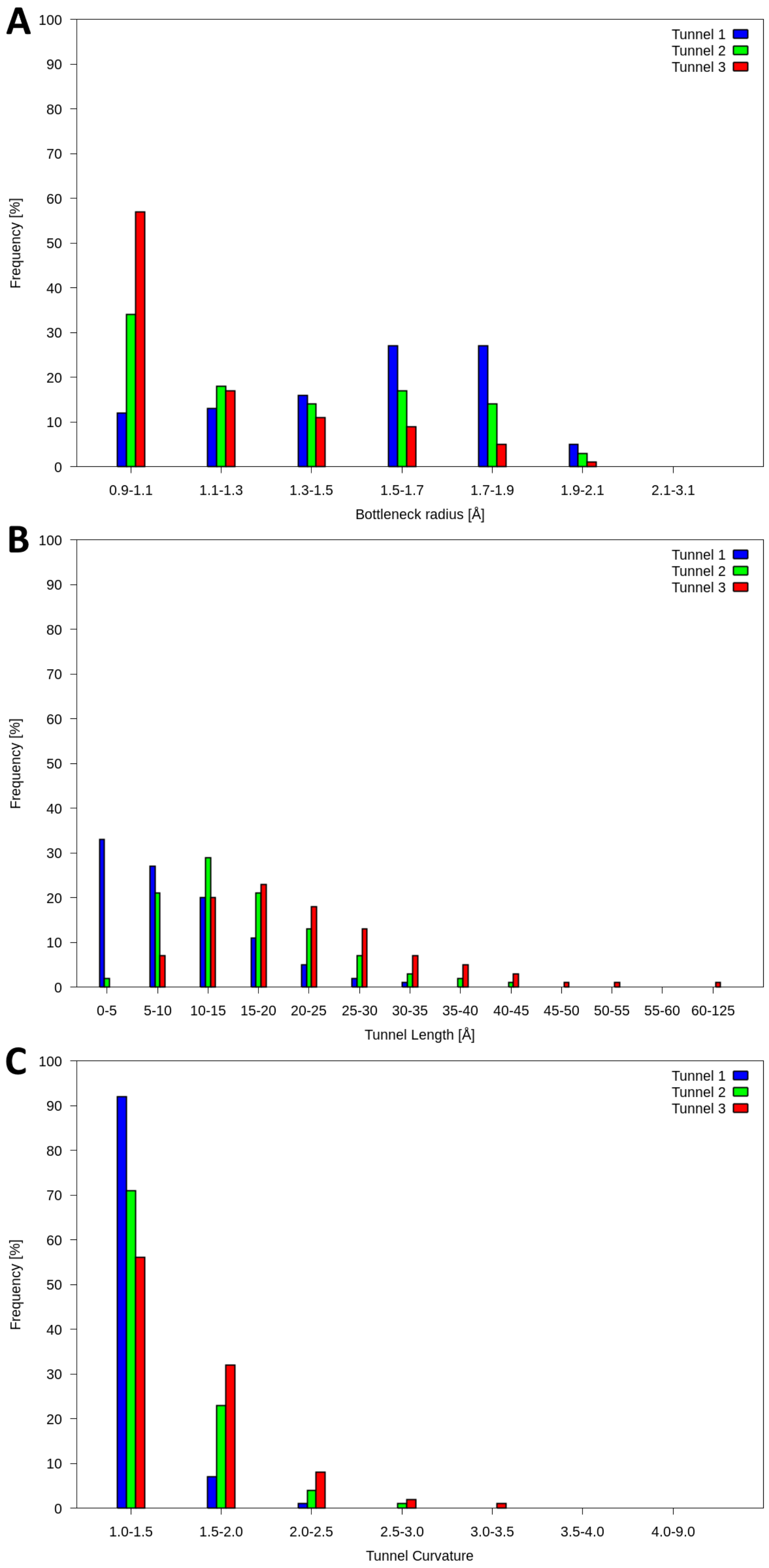

Figure S11: Distribution of tunnel parameters for first three tunnels from all tunnel cases (A) bottleneck radius, (B) tunnel length, (C) curvature.

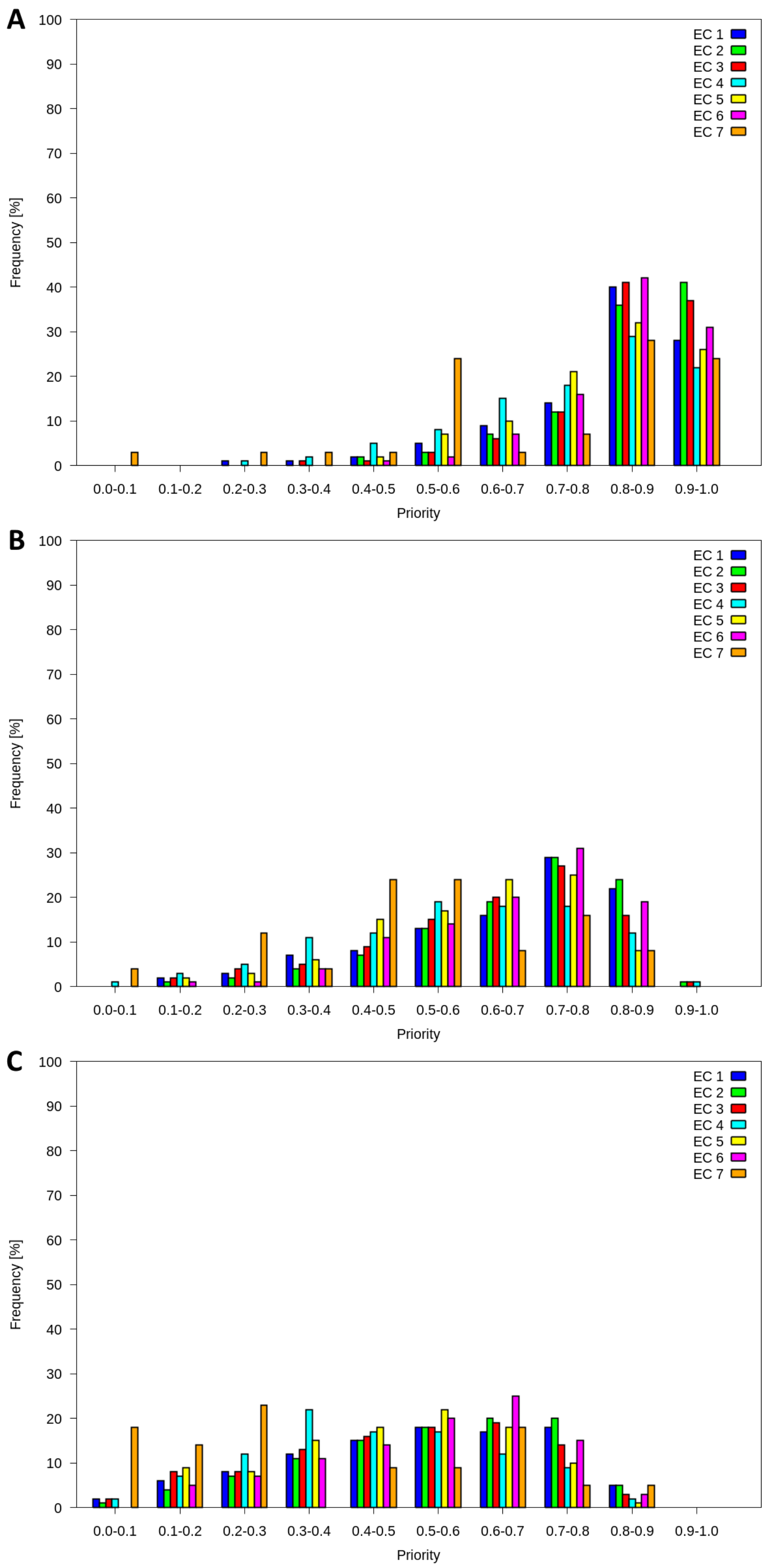

Figure S12: Distribution of tunnel priority scores in EC classes in the whole tunnel dataset for (A) tunnel 1, (B) tunnel 2, (C) tunnel 3.

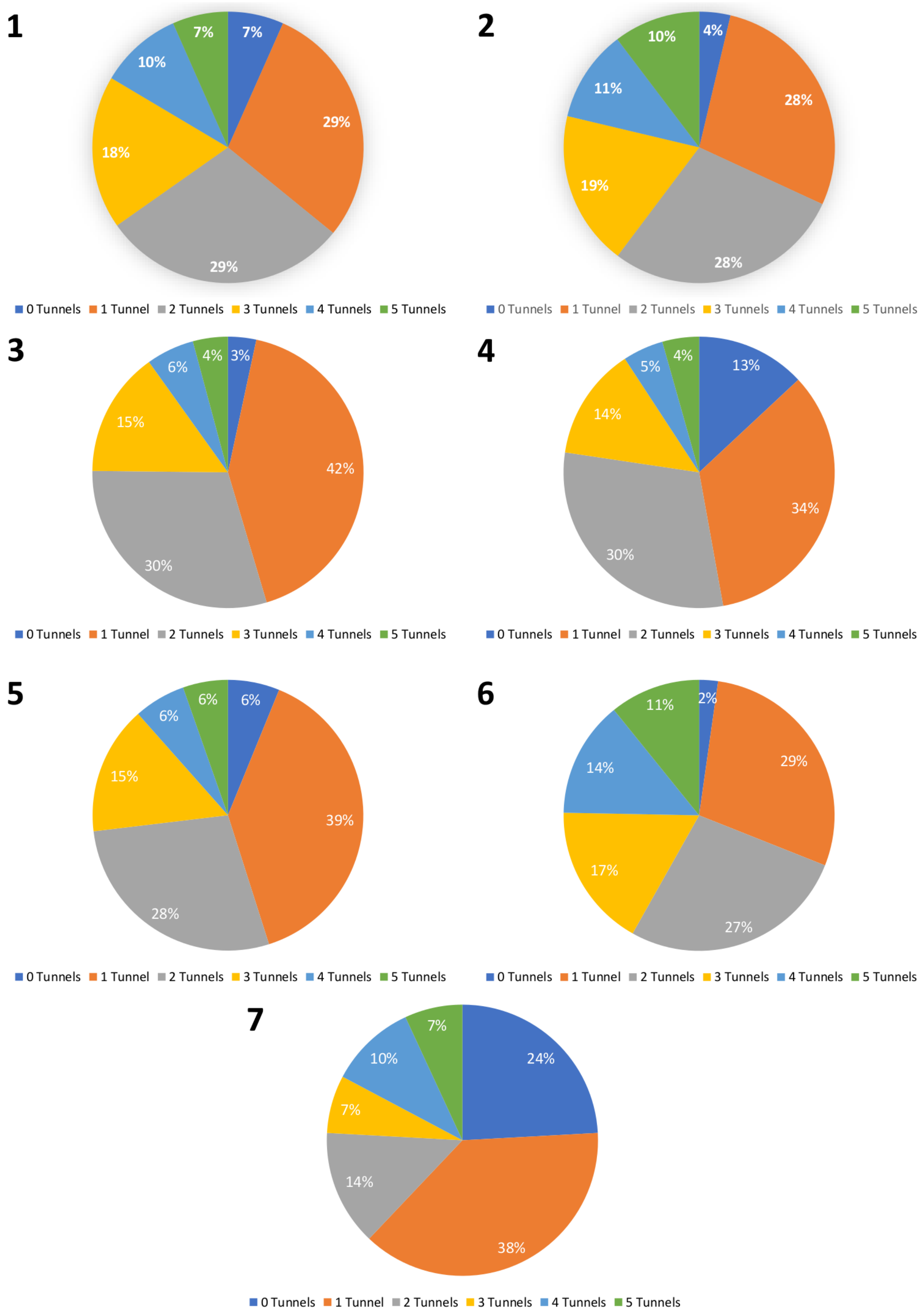

Figure S13: Number of tunnels with priority score above 0.55 found in tunnel dataset separated for each EC class (1-7).

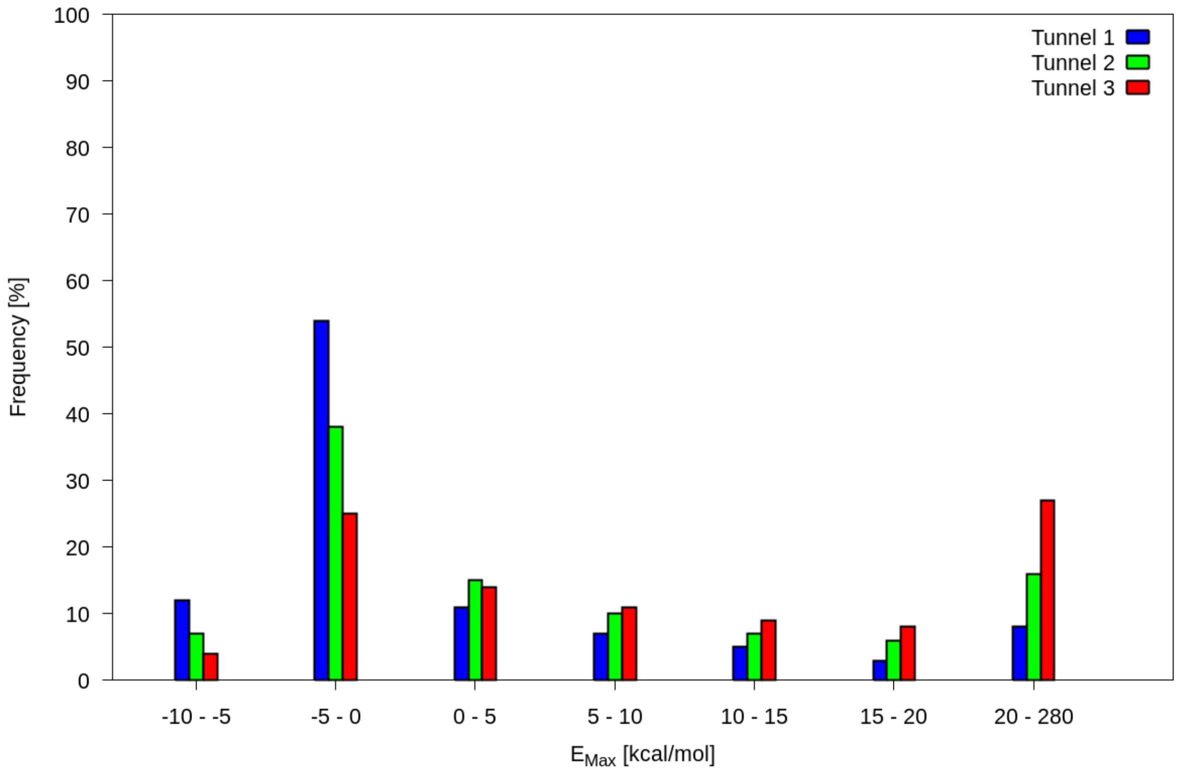

Figure S14: Distribution of energy maximum values in the CaverDock dataset for the first three tunnels. Three outliers with E_{Max} energy around -50 kcal/mol were removed from the figure.

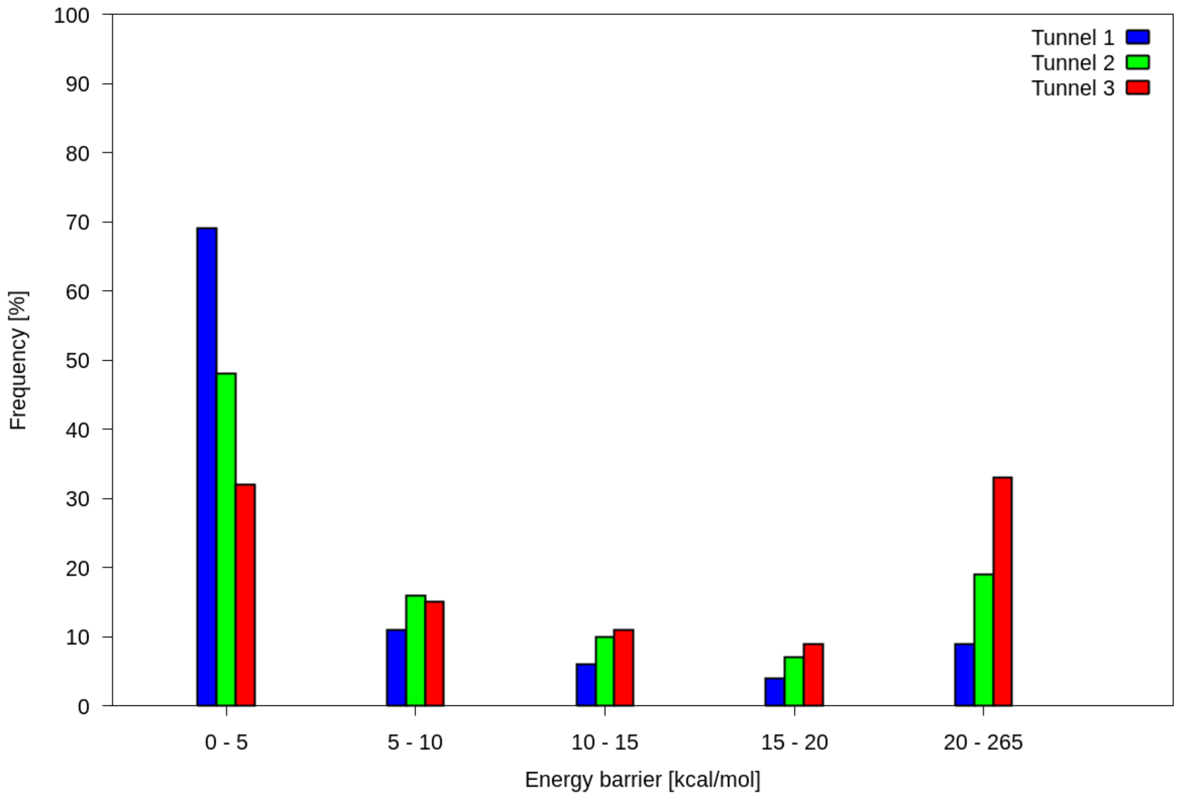

Figure S15: Distribution of energy barriers (E_a) in the CaverDock dataset for first three tunnels.
